# Kinase signalling adaptation supports dysfunctional mitochondria in disease

**DOI:** 10.3389/fmolb.2024.1354682

**Published:** 2024-01-26

**Authors:** George L. Skalka, Mina Tsakovska, Daniel J. Murphy

**Affiliations:** ^1^ School of Cancer Sciences, University of Glasgow, Glasgow, United Kingdom; ^2^ CRUK Scotland Institute, Glasgow, United Kingdom

**Keywords:** kinase, mitochondrial morphology, mitochondria, apoptosis, mitochondrial associated endoplasmic reticulum membranes (MAMs), reactive oxygen species (ROS), disease adaptation

## Abstract

Mitochondria form a critical control nexus which are essential for maintaining correct tissue homeostasis. An increasing number of studies have identified dysregulation of mitochondria as a driver in cancer. However, which pathways support and promote this adapted mitochondrial function? A key hallmark of cancer is perturbation of kinase signalling pathways. These pathways include mitogen activated protein kinases (MAPK), lipid secondary messenger networks, cyclic-AMP-activated (cAMP)/AMP-activated kinases (AMPK), and Ca^2+^/calmodulin-dependent protein kinase (CaMK) networks. These signalling pathways have multiple substrates which support initiation and persistence of cancer. Many of these are involved in the regulation of mitochondrial morphology, mitochondrial apoptosis, mitochondrial calcium homeostasis, mitochondrial associated membranes (MAMs), and retrograde ROS signalling. This review will aim to both explore how kinase signalling integrates with these critical mitochondrial pathways and highlight how these systems can be usurped to support the development of disease. In addition, we will identify areas which require further investigation to fully understand the complexities of these regulatory interactions. Overall, this review will emphasize how studying the interaction between kinase signalling and mitochondria improves our understanding of mitochondrial homeostasis and can yield novel therapeutic targets to treat disease.

## Introduction

The symbiosis between archaic mitochondria and proto-eukaryote represents a fundamental event in evolution (Martin et al., 2001; Timmis et al., 2004; Embley and Martin, 2006). The success of this milestone was dependent upon adaptation of precursor signalling pathways, a process further complicated by the transfer of mitochondrial genes into the host nuclear genome (Adams and Palmer, 2003; Roger et al., 2017). This integration into eukaryotic cells has led to the canonical symbiotic relationship, in which mitochondria are central to multiple key cellular activities such as, cell death, metabolic pathways including the Krebs cycle, lipid homeostasis, and aerobic energy generation, to name a few. The disruption of this relationship can result in mitochondrial diseases, but it can also support the progression and development of broader diseases, such as cancer and neurodegeneration. In particular, cancer requires adaptation of cells to usurp anti-tumorigenic control and sustain significant metabolic rewiring. Therefore, adaptation of mitochondrial function is key to support these phenotypes during tumour development. In addition, these mitochondrially driven mechanisms can increase treatment resistance and change intertumoral dynamics. Defining how mitochondrial function is adapted during disease and characterising the pathways which control these processes is crucial to improving our understanding of pathogenic states.

## Kinase networks

The pathways which control adaptation during disease can take many forms, from modulation of gene expression to post-translational modifications of proteins. Post-translational modification (PTM) of proteins involves the addition of moieties to target residues. There is a plethora of moieties from small methyl groups to large chains of ubiquitin and poly-ADP-ribose. However, this review will focus on the role of phosphorylation, a key PTM with well-established roles in acute and chronic response to altered cell state. The human genome codes for 538 kinases, with specific expression profiles in the different cell types of the human body (Zhang et al., 2021; Johnson et al., 2023). These kinases can be broken into families based upon substrate specificity and structural similarity. This review will focus upon how the altered signalling of four kinase pathways which regulate mitochondrial function support cancer development. These pathways are the mitogen-activated protein kinases (MAPK), Lipid secondary messenger network, cyclic- AMP (cAMP)/AMP-activated protein kinase (AMPK), and the Ca^2+^/calmodulin-dependent protein kinase (CAMK) networks. However, this review is not exhaustive and there are many more signalling networks with key functions in disease and homeostasis.

### Overview of RAS/RAF/MEK/ERK network

**FIGURE 1:**
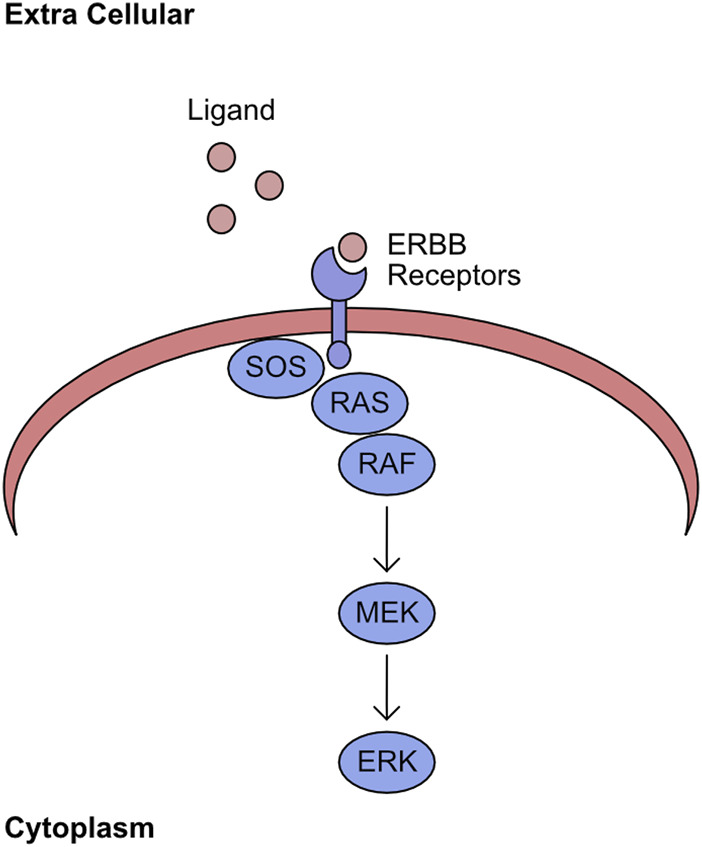
RAS/RAF/MEK/ERK pathway. Ligand binds to ERBB receptors on the plasma membrane. The intracellular domain of ligand bound ERBB receptors recruits SOS. This stabilises the GTP bound form of RAS and recruits RAF to this complex. The plasma membrane localised RAF initiates sequential phosphorylation of MEK, which in turn phosphorylates ERK.

The mitogen-activated protein kinase (MAPK) network encompasses the kinases RAF/MEK/ERK. The association of extracellular ligands to cell surface ERBB receptors activates this kinase pathway (Yarden and Sliwkowski, 2001). Upon binding to ligand, the cytoplasmic region of the receptor shifts and permits binding of the Guanine exchange factor (GEF) Son of Sevenless (SOS). This GEF promotes the exchange of GDP for GTP in the RAS binding pocket (Chardin et al., 1993). The GTP bound RAS has an increased affinity for RAF kinases, which are recruited to the plasma membrane. This re-localisation induces phosphorylation of the N-terminal auto-inhibitory region and promotes dimerization, with both events being required for full kinase activity (Mason et al., 1999; Rajakulendran et al., 2009). This initiates the sequential phosphorylation of MEK and ERK (Figure 1). The final component of this pathway, ERK, phosphorylates many targets with a wide range of cellular functions, including regulation of transcription factors (von Kriegsheim et al., 2009).

Overexpression of ERBB family members and mutation of RAS/RAF are commonly observed events in cancer. For example, ERBB2 (HER2) amplification is observed in up to 30% of breast cancers and is closely associated with poor prognosis and relapse (Slamon et al., 1987). This led to the development of a HER2 targeted monoclonal antibody, trastuzumab, which prevents ligand association with HER2 and ablates pathway activity, significantly improving patient survival (Slamon et al., 2001; Agus et al., 2002). This reliance of tumours upon ERBB signalling is also observed in lung cancer with particularly high prevalence of ERBB1 (EGFR) mutations (Zhang et al., 2016). Therefore, the current frontline therapy for advanced non-small cell lung cancer (NSCLC) are receptor tyrosine kinase inhibitors. In addition to the importance of EGFR at late stages of disease, the activity of ERBB receptors is critical during the early stages of KRAS-driven lung tumours, with the addition of tyrosine kinase inhibitors to MEK inhibition improving survival of a KRas driven murine lung cancer model (Kruspig et al., 2018; Moll et al., 2018). The mutation of Ras and RAF is commonly observed in all cancers (Prior et al., 2020; Owsley et al., 2021). Therefore, new wave drug development efforts are directly focusing on these targets. The development of inhibitors which specifically recognise mutant forms of RAS improves tumour specificity and potency (Hansen et al., 2018; Lou et al., 2019; Punekar et al., 2022). Inhibitors against MEK and BRAF are being developed and utilised, particularly in the context of melanoma (Hauschild et al., 2012; Caunt et al., 2015). The impact of pathway inhibition upon cancer is demonstrative of the critical role ERBB signalling plays in maintaining the adapted disease state.

### Overview of lipid secondary messenger network

**FIGURE 2:**
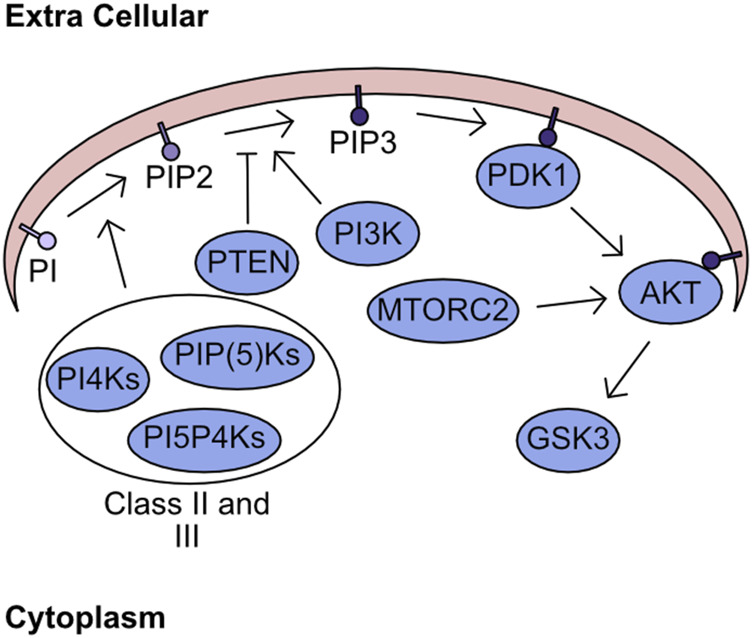
Lipid Secondary Messenger network. Phosphatidylinositol (PI) is phosphorylated by class II and class III PI kinases generating PIP2. PI3K targets PIP2 for further phosphorylation generating PIP3, which can be antagonised by the phosphatase PTEN. The presence of high concentration PIP3 regions recruits PDK1 and AKT to the plasma membrane. Sequential phosphorylation of AKT by PDK1 and MTORC2 fully activates kinase activity. Finally, the phosphorylation of GSK3 by AKT inhibits kinase activity.

The plasma membrane forms a critical barrier between the extracellular space and the intracellular milieu. This membrane is formed from a complex structured lipid bilayer, which houses numerous proteins fundamental to regulation of signalling and import/export dynamics. While these proteins are key to transferring signals from the extracellular space into the cell, the lipids themselves also have fundamental roles in cellular signalling. One group of lipids with crucial signalling activity are the phosphatidylinositols (PI), which can be phosphorylated at two positions converting PI to phosphatidylinositol-4,5-bisphosphate (PIP_2_). The two consecutive phosphorylation events are mediated through the phosphatidylinositol 4-kinases (PI4Ks), the PtdInsP-5-OH kinases (PIP(5)Ks), and the Phosphatidylinositol 5-phosphate 4-kinases (PI5P4Ks) (Whitman et al., 1987; Rameh et al., 1997; Rameh and Blind, 2023). These kinases are grouped into class II and class III. Class I refers to kinases which can targets the 3’ site and encompasses PI3K, which is responsible for phosphorylation of phosphatidylinositol-4,5-bisphosphate (PIP_2_) yielding phosphatidylinositol-3,4,5-trisphosphate (PIP_3_) (Toker and Cantley, 1997). This process of phosphorylation is not monodirectional with antagonism of this process being driven by phosphatase and tension homolog deleted on chromosome 10 (PTEN), which converts PIP_3_ into PIP_2_ (Maehama and Dixon, 1998; Myers et al., 1998; Stambolic et al., 1998). The abundance of PIP_3_ determines the activity of two kinases - 3-phosphoinositide-dependent protein kinase-1 (PDK1) and Protein kinase B (PKB/AKT). In the presence of high PIP_3_ concentration both AKT and PDK1 are recruited to the plasma membrane, through their pleckstrin homology (PH1) domains, where AKT undergoes a structural shift following association with PIP_3_ increasing catalytic activity of PDK1 towards T308 AKT (Alessi et al., 1997; Currie et al., 1999). In parallel, to support complete activation of AKT kinase activity mammalian target of rapamycin 2 (MTORC2) phosphorylates AKT S473 (Sarbassov et al., 2005). The downstream targets of AKT are numerous and overlap significantly with other members of the AGC kinase family. However, one critical target of AKT is glycogen synthase kinase-3 (GSK3) (Figure 2). GSK3 was initially described as kinase which inhibits the activity of glycogen synthase, but its functions now stretch far beyond (Embi et al., 1980). For example, it is critically involved in the regulation of cellular ROS responses through targeting of nuclear factor erythroid 2–related factor 2 (NRF2) for degradation (Rada et al., 2011).

In addition to generation of PIP_3_, PIP_2_ can also be hydrolysed by phospholipase C to form IP_3_ and diacylglycerol (DAG). The IP_3_ subsequently translocates to the endoplasmic reticulum and stimulates calcium ion (Ca^2+^) release through binding to IP3 Receptors (IP3R) (Michell and Allan, 1975; Streb et al., 1983). In addition, DAG stimulates activity of proteins containing C1 domains, such as protein kinase C (PKC) and protein kinase D (PKD) (Takai et al., 1979a; Takai et al., 1979b; Van Lint et al., 1995; Matthews et al., 1997).

The intrinsic link between PI3K/AKT/GSK3 signalling and cell growth makes it a key oncogenic driver pathway, previously reviewed in depth (Manning and Cantley, 2007; Hoxhaj and Manning, 2020). This growing evidence has further supported the initial characterisation of PTEN as a critical cellular tumour suppressor. Attempts have been made to suppress the overactivity of this pathway in cancer. However, the development of PI3K inhibitors has been hampered by the toxicity associated with candidate compounds (Ellis and Ma, 2019). While inhibition of AKT has been trialled multiple times with low toxicity. However, has shown poor clinical efficacy as a monotherapy (Hua et al., 2021).

### Overview of PKA/cAMP/AMPK network

**FIGURE 3:**
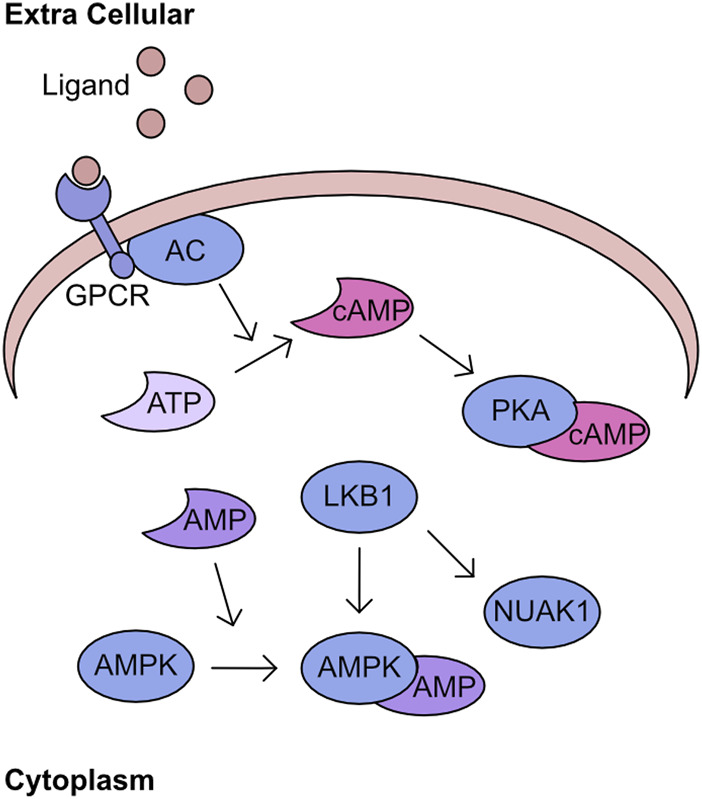
PKA/cAMP/AMPK network Activation of G-protein coupled receptors (GPCR) by extracellular ligand stimulates conversion of ATP to cAMP by adenyl cyclase (AC). This cAMP bins to PKA and stimulates kinase activity. In parallel, AMP can bind to AMPK inducing a structural change which permits phosphorylation by LKB1. This phosphorylation activates AMPK activity and is an activation mechanism conserved across the AMPK related kinase family (ARK), including NUAK1.

Recognition of cellular energy perturbations is required to support cellular viability. The fundamental molecules of the intracellular energy system are AMP, ADP, and ATP. Therefore, multiple pathways have evolved to monitor levels are these molecules. One such pathway is centred on AMPK, which is a heterotrimeric complex comprising a catalytic subunit, α, and two regulatory subunits, β and γ. The kinase activity of this complex is stimulated by binding of AMP and ADP which induces a structural change permitting phosphorylation of T172 by Liver kinase B1 (LKB1) to fully activate kinase activity (Figure 3) (Yeh et al., 1980; Carling et al., 1987; Oakhill et al., 2011; Xiao et al., 2011). Activated AMPK inhibits pathways such as gluconeogenesis and translation, while promoting catalytic processes including increased glucose utilisation and mobilisation of lipid stores. The combined activity of these regulatory cascades supports the restoration of cellular ATP generation. A comprehensive overview of the field’s history can be found in these two reviews (Hardie et al., 2011; Mihaylova and Shaw, 2011). Importantly, AMPK is the archetypical member of the AMPK related kinase (ARK) family, which consists of 12 other members all of which are activated by LKB1 mediated phosphorylation at a T-loop threonine (Lizcano et al., 2004). An overview of the functions of the kinases within this family can be found in these two reviews (Bright et al., 2009; Monteverde et al., 2015). One of the kinases most closely related to AMPK is NUAK1, which has a diverse set of functions including regulation of centrosome number, MYC-dependent splicing control, HIPPO signalling control, and importantly cellular ROS responses (Humbert et al., 2010; Port et al., 2018; Cossa et al., 2021; Whyte et al., 2023). The phosphorylation of MYPT at S445 by NUAK1 increases the activity of GSK3β for NRF2 and suppresses the cellular ROS response thereby reducing the capacity of cancer cells to maintain adaptation to high ROS production (Port et al., 2018). In addition, the over-expression of NUAK1 in the cytoplasm supports glycolytic switch of cancer cells (Escalona et al., 2020). As demonstrated by LKB1/AMPK activation following increased AMP levels, these nucleotides have critical signalling capacity. A further signalling role for these nucleotide molecules is through the adenylyl cyclases (AC). There are ten AC found within the human cell, nine of which are membrane bound and one which is cytoplasmic. These generate cAMP by conversion of ATP following the activation of G-protein coupled receptors (GPCRs) (Scholich et al., 1999; Pierce et al., 2002). Importantly, the generation of cAMP is spatially regulated, generating microdomains of signalling (Zaccolo and Pozzan, 2002; Pierre et al., 2009). The increased cAMP activates protein kinase A (PKA), which phosphorylates a multitude of downstream targets (Figure 3) (Corbin and Krebs, 1969).

Given the roles of AMPK signalling in regulation of energy homeostasis this pathway has major roles underpinning cancer cell viability (Hardie, 2013). A recent example demonstrates the importance of AMPK during prostate cancer development through control of catabolic signalling (Penfold et al., 2023). Following previous work which found increased sensitivity of LKB1 deficient lung cancers due to the rewiring of AMPK signalling (Shackelford et al., 2013). The functional importance of the ARK family for cancer persistence is not limited to AMPK. The loss of NUAK1 expression induces apoptosis in cancer cells with over-expression of MYC, a commonly observed phenotype of tumorigenesis (Liu et al., 2012).

### Overview of calcium kinase network

**FIGURE 4:**
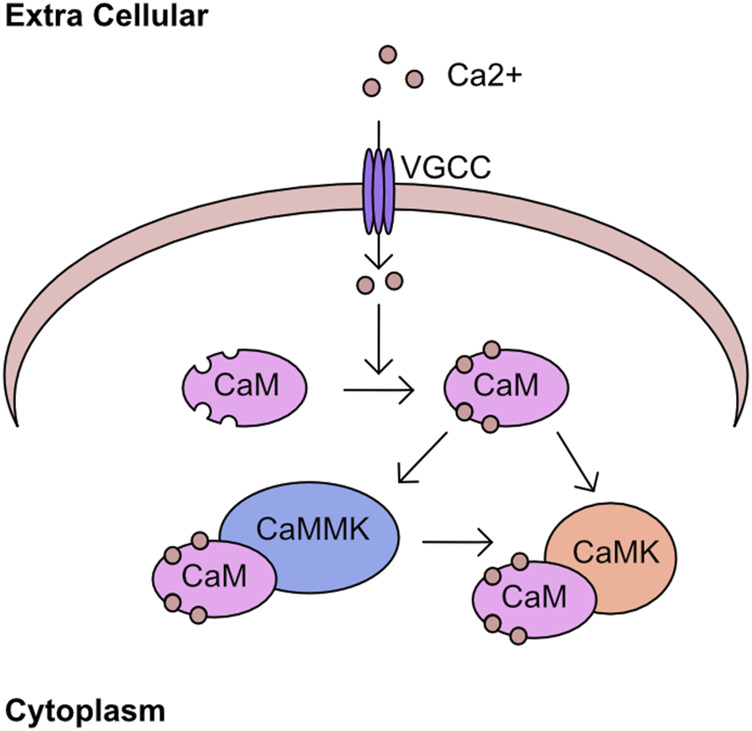
Calcium kinase network Calcium enters the cell through cell surface channels, including the voltage gated calcium channels (VGCC). Once in the cytoplasm calcium associates with calmodulin (CaM) through four EF hand domains. The calcium bound CaM binds to calcium/calmodulin-dependent kinase kinase (CAMKK2) and stimulates kinase activity toward downstream targets including calcium/calmodulin-dependent protein kinase (CAMK).

The regulation of cellular calcium influx is most closely associated with neuronal cell function and transcriptional responses, with the major family of import channels referred to as voltage gated Ca^2+^ channels (VGCC) (Hardingham et al., 1997; Catterall, 2011). However, the regulation of cellular calcium in non-neuronal cell types is equally critical (Berridge et al., 2003; Berridge, 2012; Bootman and Bultynck, 2020). One pathway of calcium signalling is driven through the calcium/calmodulin associated kinases (CaMK) and phosphatases. The calcium binding protein calmodulin is a key component of this pathway, containing 4 EF-hand domains capable of binding calcium and driving association with binding partners (Means and Dedman, 1980; Babu et al., 1985). These binding partners include the CaMK kinases which has two isoforms α (CAMKKα) and β (CAMKKβ), or through binding to CAMK kinase 2 (CAMKK2). These kinases phosphorylate a threonine residue on the activation loop of calcium/calmodulin kinases (CaMK) and permit full catalytic activation in combination with binding to calmodulin (Figure 4). An overview of the early discoveries and regulatory steps in these pathways was recently published (Marcelo et al., 2016). Interestingly, in addition to regulation of CAMKI and CAMKIV, CAMKK2 was also found to activate AMPK in certain tissues or following loss of LKB1 (Woods et al., 2005; Green et al., 2011; Mairet-Coello et al., 2013).

The perturbation of calcium signalling is associated with the onset of various neurodegenerative diseases (Mattson, 2007). However, the involvement of the downstream kinases is understudied by comparison. The loss of CAMKK2 expression has been associated with altered transferrin regulation which can drive over production of ROS (Sabbir, 2018). The loss of CAMKK2 activity also protects against the synaptoxicity of Amyloid β in murine *in vitro* (Thornton et al., 2011; Mairet-Coello et al., 2013). In the context of cancer CAMKK2 has been associated with development of prostate cancer, activation of AKT signalling in ovarian cancer, and regulation of immune microenvironment in breast cancer (Gocher et al., 2017; Penfold et al., 2018; Racioppi et al., 2019).

## Mitochondrial function is controlled by PTMs and supports disease

This review will focus on four areas of mitochondrial biology and how these processes are deregulated by altered kinase signalling in disease: mitochondrial morphology, apoptosis, calcium homeostasis, and reactive oxygen species (ROS) generation. A recent key investigation has demonstrated the importance of phosphorylation for metabolic control (Geffen et al., 2023). However, the impact of PTM on mitochondrial metabolism has been extensively reviewed elsewhere (Humphrey et al., 2015; Kitamura and Galligan, 2023).

### Mitochondrial morphology

The mitochondrial network is highly dynamic and, in response to various intracellular and extracellular signalling events, can change morphological state. Mitochondria are either fused into an elongated continuous network or undergo fission to compartmentalise mitochondria into punctate units. The proteins that regulate this process can be split into two groups: the pro-fission proteins and pro-fusion proteins. The phosphorylation of proteins involved in the processes of mitochondrial fusion and fission has major consequences for protein function. However, do these phosphorylation events have the capacity to support the development of disease states and is it a necessary step during cellular adaptation?

### Mitochondrial fission

The three major regulators of mitochondrial fission are Dynamin related protein 1 (DRP1), fission protein 1 (FIS1), and mitochondrial fission/fusion factor (MFF). When mitochondrial fission is initiated DRP1 is recruited to the mitochondrial membrane bound receptors MFF and FIS1 (James et al., 2003; Stojanovski et al., 2004; Gandre-Babbe and van der Bliek, 2008; Otera et al., 2010). A DRP1 oligomer encircles the mitochondria and through dynamin activity cleaves through the mitochondrial membrane (Mears et al., 2011; Kalia et al., 2018). The phosphorylation of these proteins has a major impact upon activity and will define mitochondrial fission/fusion status in multiple scenarios. The importance of DRP1 serine 616 (S616) and serine 637 (S637) for controlling the activity of DRP1 during cell cycle, cell death, and mitophagy induction will now be covered. In general, phosphorylation of DRP1 S616 is associated with increased mitochondrial fission and phosphorylation of S637 is associated with fusion. During mitosis, DRP1 associates with the RALA/RALBP1 complex and is phosphorylated at S616 by the CDK1/cyclin B complex, supporting fission of the mitochondrial network (Kashatus et al., 2011). Loss of DRP1 expression or over-expression of a non-phosphorylatable form suppressed this mitochondrial fragmentation and antagonised progression through mitosis (Taguchi et al., 2007). Following the stimulation of cells with growth factors, the phosphorylation of DRP1 at S616 by ERK drives mitochondrial fission, which can be blocked by the application of MAPK inhibitors (Kashatus et al., 2015; Serasinghe et al., 2015). The morphological state of the mitochondrial network will influence the capacity of cells to induce apoptosis. In cardiac cells the chronic activation of β-adrenergic receptors (β-AR) over-activates CaMKKII which directly phosphorylates DRP1 at S616. The increased phosphorylation supports induction of cell death and cardiac damage, which can be reversed by the application of CAMKKII inhibitors (Xu et al., 2016). In contrast, the phosphorylation of DRP1 at S637 by PKA drives mitochondrial fusion and increases resistance to cell death following treatment with apoptosis inducers. This phenotype can be reverted by overexpression of a non-phosphorylatable DRP1 S637A mutant (Chang and Blackstone, 2007; Cribbs and Strack, 2007). Interestingly, the increased expression of dominant negative mutant of Rab32 T39N can increase phosphorylation of S637 through regulation of PKA recruitment to mitochondria (Bui et al., 2010). The induction of mitophagy is an important process which permits clearance of unwanted mitochondria. This requires encapsulation of mitochondria by auto-phagosome membranes (Palikaras et al., 2018). The phosphorylation of DRP1 at S616 by PINK1 kinase drives mitochondrial fission and supports mitophagy (Han et al., 2020). Phosphorylation events with defined phosphorylation sites mediating mitochondrial morphology are summarised in Table 1. The stability of phosphorylation events is defined by a balance between the activity of kinases and phosphatases. The phosphatase Calcineurin dephosphorylates the S637 site of DRP1 and regulates its translocation to mitochondria inducing mitochondrial fusion (Cereghetti et al., 2008; Sandebring et al., 2009).

**TABLE 1:** Phosphorylation events regulating mitochondrial network dynamics. AMPK, AMP-activated protein kinase; CaMKIα, calcium/calmodulin-dependent protein kinase Iα; CDK1, cyclin-dependent kinase 1; DRP1, dynamin related protein 1; ERK, extracellular signal-regulated kinase; GSK3β, glycogen synthase-3 beta; FIS1, fission protein 1; MET, mesenchymal-epithelial transition factor; MFF, mitochondrial fission/fusion factor; MFN2, mitofusin 2; PINK1, PTEN induced kinase 1; PKA, protein kinase A; PKD, protein kinase D.

Target	Phospho site	Kinase	Associated outcome
DRP1	S600	CaMKIα	Translocation to mitochondria
DRP1	S616	CDK1/cyclin B complex,ERK, CaMKKII, PINK1	Increased mitochondrial fission
DRP1	S637	PKA	Increased mitochondrial fusion
DRP1	S693	GSK3β	Resistance to oxidation-induced mitochondrial network fragmentation
DRP1	S40, S44 (in neuronal cells)	GSK3β	Increased mitochondrial fragmentation
FIS1	Y38	MET	Increased mitochondrial fission
MFF	S129, S146	AMPK	Increased mitochondrial fission
MFF	S155, S172, S275	PKD	Increased mitochondrial fission in mitosis
MFN2	T111, S442	PINK1	Mitophagy induction

In addition to the phosphorylation of S616 and S637 multiple alternative sites also define DRP1 function. Phosphorylation of DRP1 by CaMKIα at S600 increases the translocation to mitochondria, mediated by an increased affinity for FIS1 (Han et al., 2008). The phosphorylation of DRP1 at S693 by GSK3β can drive resistance to H_2_O_2_ induced fragmentation of the mitochondrial network and apoptosis induction, which is further recapitulated by overexpression of S693D mutant DRP1 (Chou et al., 2012). In addition to phosphorylation at S693, GSK3β also phosphorylates S40 and S44 sites in neuronal cells, this can drive increased GTPase activity and increase fragmentation of the mitochondria, this is also driven by phospho-mimetic mutants at S40 and S44 (Yan et al., 2015).

The majority of investigations have focused upon DRP1 phosphorylation events in isolation. However, the interplay between these modifications poses an intriguing question. For example, the phosphorylation of S600 enhances phosphorylation at S579, with both of these sites required for complete mitochondrial fragmentation. Interestingly, loss of S600 induces disrupted mitochondrial morphology and metabolic capacity in mice carrying a S600A mutation in DRP1 (Valera-Alberni et al., 2021). This study suggests that further investigation of the interplay between phosphorylation sites is critical to our complete understanding of mitochondrial morphology regulators.

**FIGURE 5:**
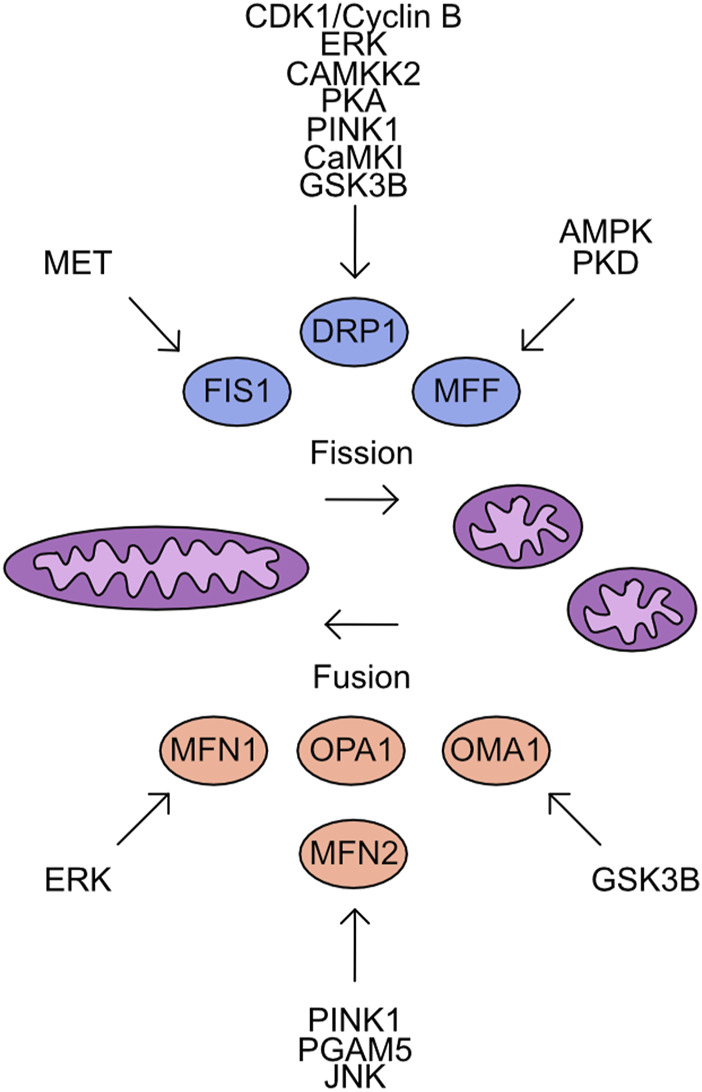
Overview of kinases which target the mitochondrial Fusion/Fission machinery. Mitochondrial fission is driven by the activity of FIS1, DRP1, and MFF. In contrast, mitochondrial fusion is supported by MFN1, MFN2, OPA1, and OMA1. Shown are the identified kinases which target these proteins.

These studies demonstrate the nuanced regulatory system that controls DRP1 activity which is cell type and cell state specific. A further complicating factor is the parallel phosphorylation of other factors which control mitochondrial fission/fusion dynamics. Much like DRP1 both FIS1 and MFF are regulated by phosphorylation events. FIS1 phosphorylation at Y38 by MET drives mitochondrial fission through increased recruitment of DRP1 to the mitochondrial outer membrane in hepatocellular carcinoma cell lines (Qi et al., 2016). MFF phosphorylation at S129 and S146 by AMPK and drives mitochondrial fission responses (Ducommun et al., 2015; Toyama et al., 2016). Protein kinase D (PKD) targets MFF S155, S172, and S275, with loss of phosphorylation at these sites driving defects in mitochondrial fission and leads to abnormal chromosome segregation during mitosis (Figure 5) (Pangou et al., 2021).

### Mitochondrial fusion

The antagonists to mitochondrial fission are the proteins which control mitochondrial fusion. The process of mitochondrial fusion requires proteins to act at both the outer and inner mitochondrial membranes. The outer membrane proteins are Mitofusin 1 (MFN1) and Mitofusin 2 (MFN2) which drive fusion of adjacent mitochondrial membranes through GTPase activity (Ishihara et al., 2004; Qi et al., 2016; Li et al., 2019). Both proteins are subject to multiple phosphorylation events which impact their functional activity. Phosphorylation of MFN1 by ERK at T562 reduces mitochondrial fusion and instead promotes BAK association with the mitochondrial membrane more efficiently, inducing apoptosis (Pyakurel et al., 2015). MFN2 is phosphorylated by PINK1 at T111 and S442 and triggers parkin association with the mitochondria inducing mitophagy (Chen and Dorn, 2013; Li et al., 2022). The dephosphorylation of MFN2 S27 by Mitochondrial Serine/Threonine protein phosphatase (PGAM5) protects MFN2 from degradation (Nag et al., 2023). In contrast, the JNK dependent phosphorylation of MFN2 recruits ubiquitination complexes which degrade MFN2 and increase the cellular sensitivity to apoptosis (Figure 5) (Leboucher et al., 2012).

The regulation of mitochondrial inner membrane fusion is controlled by the cleavage of OPA1 (Mishra et al., 2014). While phosphorylation moieties have been detected on OPA-1 no upstream kinases have been characterised. The cleavage of OPA-1 is regulated by OMA-1. Phosphorylation of OMA-1 by GSK3β has been detected and is critical during oocyte development (Nishi and Lin, 2005; Shirayama et al., 2006; Stitzel et al., 2006).

### Mitochondrial fission and fusion protein modification supports disease

The perturbed function of fission and fusion proteins underpins the development of many diseases. For example, DRP1 function is critical for the development of RAS driven skin, pancreas, brain, squamous cell carcinoma, and colorectal cancers, with loss of expression preventing cellular transformation (Serasinghe et al., 2015; Xie et al., 2015; Kitamura et al., 2017; Nagdas et al., 2019; Sessions et al., 2022; Zhu et al., 2022). Interestingly, the development of lung adenocarcinoma seems independent of DRP1 but is instead dependent upon the expression of OPA1 (Sessions et al., 2022). Two recent reviews provide a further exploration of fusion/fission protein function in disease (Serasinghe and Chipuk, 2017; Yapa et al., 2021). However, how does the alteration to specific phosphorylation sites, rather than total protein function, drive disease?

Many studies have focused on the phosphorylation of DRP1 and its role in tumorigenesis. Interestingly the S616 and S637 sites seem to act as a regulatory convergence point for the kinase pathways outlined earlier. Firstly, the phosphorylation of DRP1 at S616 is required for the transformation of melanocytes cells which overexpress RAF. However, even in the absence of RAF the over-expression of constitutively phosphorylated DRP1 is sufficient to support transformation of melanocytes (Serasinghe et al., 2015). This provided strong evidence of DRP1's capacity to drive oncogenic transformation of cells. This is further supported by multiple reports of ERK mediated DRP1 S616 and S637 phosphorylation driving cellular transformation. For example, S616 phosphorylation supports mitochondrial functionality in lung cancer and promotes proliferation and migration of tumour cells (Chung et al., 2021). While the phosphorylation of DRP1 at S637 by ERK is required for the generation of inducible-pluripotent stem cells in tumours (Prieto et al., 2016). Interestingly, the nucleotide dependent kinase pathways also target the S616 and S637 sites. Salt inducible kinase 2 (SIK2) phosphorylates DRP1 S616 in ovarian cancer cells and therefore promotes mitochondrial fission, supporting tumour cell survival (Han et al., 2020). The PKA mediated phosphorylation of DRP1 S637 is required for hepatocellular carcinoma cell response to energy stress. This was driven by elongation of the mitochondrial network, increased cristae formation, and a switch towards oxidative phosphorylation, this could be reversed by overexpression of DRP1 S637A mutants (Li et al., 2017).

In addition to these early events in tumorigenesis, the modulation of S616 phosphorylation has also been associated with adaptation of tumours towards cancer therapies. Firstly, mitochondrial dynamics are associated with the resistance of tumour cells to chemotherapy. The induction of DRP1 mediated mitochondrial fragmentation and glycolytic switch in T cell acute lymphoblastic leukaemia co-cultured with mesenchymal stem cells drives chemoresistance (Cai et al., 2016). The activation of ERK signalling through HMGB1 and activation of RAGE pathway induced phosphorylation of DRP1 S616 and drove autophagy increasing chemoresistance of colorectal cell lines (Huang et al., 2018). Secondly, the exposure of tumours to ionising radiation (IR) is a common front-line treatment. However, the impact of IR on the mitochondria is poorly understood. One study identified CAMKKII as driving phosphorylation of DRP1 S616, which supports network fragmentation in response to IR (Bo et al., 2018). Finally, the treatment with hormonal therapy is common for cancers, such as, breast and ovarian. The resistance of tumours to these treatments is a key problem, and the cellular mechanisms which drive this process need further investigation. Interestingly, tamoxifen resistant breast cancer cell lines showed a decreased respiratory capacity, increased superoxide, and mitochondrial fission with increased DRP1 S637 phosphorylation (Tomkova et al., 2019). The identification of increased S637 in fragmented mitochondrial networks was intriguing, as this site is usually associated with fusion. Therefore, more work will be required to establish the kinase pathway which drives this phosphorylation and if this necessary for tamoxifen resistance.

The phosphorylation of DRP1 has been studied in multiple contexts and supports the adaptation of cells during the development of disease, such as cancer. However, the regulation of other fusion and fission family members by PTMs is greatly understudied and the potential for this process to underpin disease development is significant. This is demonstrated by the PINK1 mediated phosphorylation of MFN2 S442 which promotes its degradation and supports proliferation of lung cancer cell lines (Dasgupta et al., 2021).

### Targeting mitochondrial morphology in disease

The evidence for mitochondrial fusion and fission protein phosphorylation supporting disease has driven research to pharmacologically target these events in disease. Despite the well-characterised role of mitochondrial fusion and fission proteins in cancer development and progression no clinical trials have explored targeting mitochondrial morphology as a viable anti-cancer therapeutic strategy. Moreover, clinical and preclinical trials exploring kinase inhibition in advanced models of cancer, for example, ERK and MEK inhibition, did not characterise downstream effects on mitochondria morphology and function (Caunt et al., 2015; Moschos et al., 2018). However, clinical intervention targeting mitochondrial kinases has been investigated in neurodegeneration and cardiac ischemia. The inhibition of ERK restores the balance between mitochondrial fusion and fission to repair aberrant mitochondrial morphology in hybrid neurons with incorporated platelet mitochondria from Alzheimer’s disease patients highlighting the promise behind ERK targeting (Gan et al., 2014). Importantly, the astragaloside IV derivative LS-102 alleviated myocardial ischemia reperfusion injury in H9c2 cells by blocking GSK3β-mediated DRP1 phosphorylation at S616 and hence mitochondrial fragmentation in a similar manner to the GSK3β inhibitor AR-A014418 (Chen et al., 2020).

The clinical benefits of targeting mitochondrial morphology can be further illustrated by trials in cardiovascular disease. A phase II clinical study investigates Sovateltide (PMZ-1620; IRL-1620) for the treatment of hypoxic-ischemic encephalopathy (HIE) (NCT05514340). Sovateltide is an endothelin B receptor (ETBR) agonist promoting higher differentiation of neuronal progenitor cells with improved mitochondrial morphology and thus function (Ranjan et al., 2020). The above investigation follows from Sovateltide’s first approval in May 2023 in India for the treatment of cerebral ischemic stroke within 24 h of stroke onset (Keam, 2023). Evidence of Trimetazidine cardioprotective effects via improved balance between mitochondrial fusion and fission underpinned a phase II clinical trial investigating Trimetazidine’s effects on patients with pulmonary hypertension (PAH) (Shi et al., 2017) (NCT02102672). The investigators had hypothesised that fatty acid oxidation inhibition by Trimetazidine will restore mitochondrial morphology and function in PAH patients. In general, preclinical therapeutic strategies aimed at supporting healthy mitochondrial networks including DRP1 inhibition and MFN activation were deemed central to cardioprotection (Hernandez-Resendiz et al., 2023). These data point to the potential behind kinase targeting to modulate mitochondrial networks in diseases presenting with aberrant mitochondrial morphology.

Nevertheless, the number of clinical trials targeting mitochondrial morphology control both through kinase regulation and directly is still low highlighting an unmet need.

## Regulation of cellular apoptosis

**FIGURE 6:**
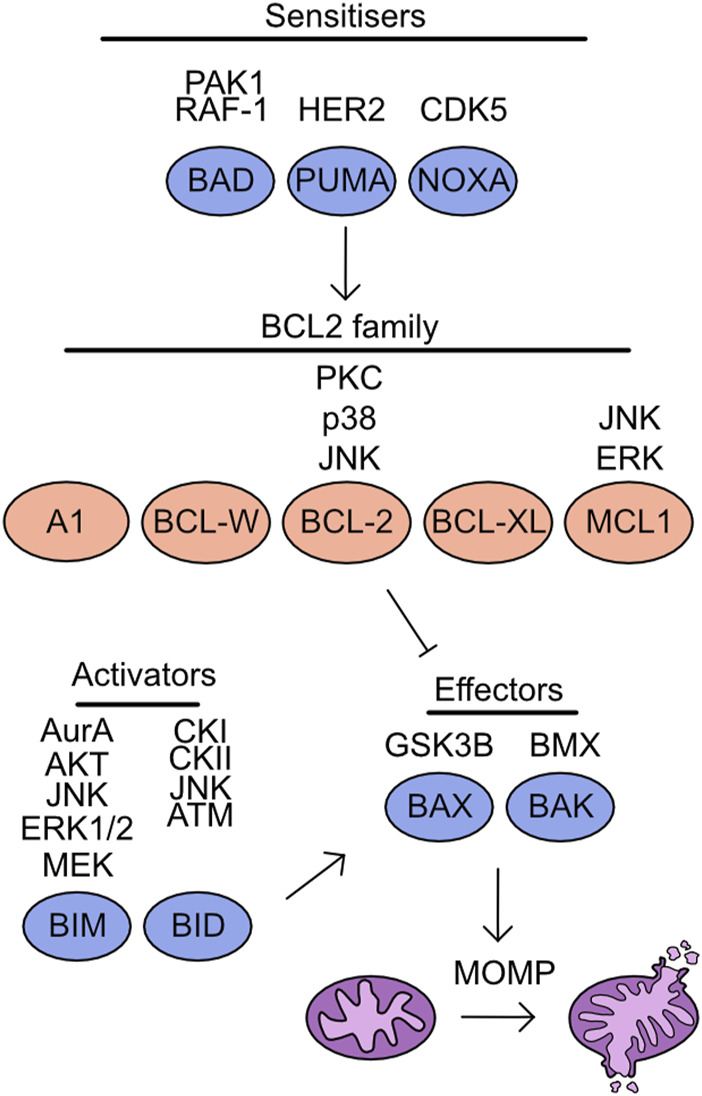
Overview of kinases which target the cell death machinery. Mitochondrial apoptosis is driven through the activity of effector molecules BAX/BAK, which form oligomeric pores on the outer mitochondrial membrane initiating mitochondrial outer membrane permeabilization (MOMP). The induction of MOMP is suppressed by the association of BCL2 family proteins to BAX/BAK. However, in the presence of either sensitisers reducing BCL2 family binding to BAX/BAK or through direct activity of apoptotic activators MOMP is induced. Listed above are the identified kinases which target each member of this pathway.

Multiple mechanisms of cell death have been characterised, including apoptosis, pyroptosis, necroptosis, and ferroptosis. The regulation of apoptosis can be broken into intrinsic and extrinsic pathways. The mitochondria play a key role during induction of intrinsic apoptosis, which is tightly regulated by the BCL-2 homology domain (BH) family proteins. These proteins are characterised by the presence of BH domains and can be functionally separated into anti-apoptotic and pro-apoptotic. The anti-apoptotic family proteins include: BCL-2 related gene A1 (A1), BCL-w, BCL-2, BCL-2 long isoform (BCL-XL), and myeloid cell leukaemia 1 (MCL-1) (Vitale et al., 2023) (Figure 6). These proteins contain four BH domains, are predominantly localised to the outer mitochondrial membrane and bind to BH3 only proteins suppressing apoptosis induction (Llambi et al., 2011). In addition, a limited role of these proteins has been identified during pyroptosis and necroptosis induction. BCL‐2 associates with BH3-like domains in key regulators of these cell death pathways, gasdermin D (GSDMD) and mixed lineage kinase domain-like (MLKL). This increases cleavage of GSDMD at S87 and limits activation of MLKL by RIP3, thereby reducing induction of pyroptosis and necroptosis (Shi and Kehrl, 2019).

The remaining BH family members are classed as BH3 only proteins and are pro-apoptotic, this includes BAX, BAK, BIM, BID, BAD, p53 upregulated modulator of apoptosis (PUMA), and phorbol-12-myristate-13-acetate-induced protein 1 (NOXA) (Vitale et al., 2023) (Figure 6). Both BAX and BAK are “effectors” as these proteins can directly associate with the outer mitochondrial membrane and induce mitochondrial outer membrane permeabilization (MOMP). The importance of these proteins for apoptosis induction can be demonstrated by the inability of BAX/BAK deficient cells to undergo apoptosis (Wei et al., 2001). The remaining pro-apoptotic family members are split into two groups, the “activators” are BIM and BID and can either interact with BCL-2 family members or activate BAX/BAK directly, either in apoptotic induction or to support necroptosis (Tischner et al., 2012). While “sensitisers”, BAD, PUMA, and NOXA bind to the anti-apoptotic family members and decrease the threshold for MOMP induction. For a comprehensive overview of the literature surrounding these fundamental proteins and their discovery, see these reviews (Chipuk et al., 2010; Delbridge and Strasser, 2015). Following MOMP induction cytochrome C is released into the cytoplasm, promoting caspase cleavage and further apoptotic processes (Kluck et al., 1997; Li et al., 1997). Increasing evidence is emerging of limited and non-apoptotic MOMP which can lead to multiple cellular consequences from induction of inflammation to apoptosis resistance (Killarney et al., 2023).

The induction of extrinsic apoptosis is characterised by association of ligands, such as FasL, TRAIL, or TNFα to cell surface receptors, a contrast to intrinsic apoptotic processes (Itoh et al., 1991; Oehm et al., 1992; Wiley et al., 1995; Pitti et al., 1996). The binding of ligand drives formation of the death inducing signalling complex (DISC) which promotes cleavage and activation of caspase 8 (Kischkel et al., 1995). This activated caspase 8 directly cleaves caspase 3/7 further promoting apoptosis induction, while the mitochondrial apoptotic cascade is activated in parallel (Scaffidi et al., 1998; Wachmann et al., 2010). Importantly, these ligands can also induce necroptotic pathways. The binding of Fas can induce caspase independent cell death of T cells (Holler et al., 2000). While the inhibition of caspase-8 activation following TNF or TRAIL mediated pathway activation can drive initiation of necroptosis through RIPK3 and MLKL (Vercammen et al., 1998; Zhang et al., 2009).

The regulation of apoptotic proteins by phosphorylation has been characterised in multiple settings and can have significant impact upon the response of these proteins to apoptotic signals.

### BAD

BAD binds to BCL-2 family members, thereby preventing these proteins from associating with BAX/BAK (Yang et al., 1995). The capacity of BAD to bind with BCL-2 family members is regulated by phosphorylation. The phosphorylation of S112 and S136 is mediated by AKT in response to growth factor availability and suppresses apoptosis induction (del Peso et al., 1997; Datta et al., 1997). The phosphorylation of these two sites in combination with a further phosphorylation at S155 promotes association with 14-3-3 proteins and degradation of BAD (Datta et al., 2000). The S112 site was found to be particularly promiscuous, with ribosomal s6 kinase (RSK), PKA, and Raf-1 also capable of targeting this site (Bonni et al., 1999; Harada et al., 1999; Polzien et al., 2009). Further to these previously identified sites, the phosphorylation of S111 by PAK1 has also been identified, as with the S112 site this supports association with 14-3-3 proteins and promotes cell survival through availability of BCL-2 proteins (Polzien et al., 2009; Ye et al., 2011).

### BID

BID is a BH3-only protein which is cleaved by caspase activity following activation of extrinsic apoptosis signals (Li et al., 1998; Luo et al., 1998). Cleaved BID, known as tBID, subsequently translocates to the mitochondrial outer membrane and induces BAX/BAK activity to drive MOMP (Gross et al., 1999; Eskes et al., 2000; Wei et al., 2000). The phosphorylation of BID at S61 and S64 by casein kinase I (CKI) and casein kinase II (CKII) suppress apoptosis induction by FAS ligand, through reduced cleavage of Bid by caspase 8 (Desagher et al., 2001; Degli Esposti et al., 2003). In contrast, a study identified phosphorylation of BID by JNK at T59 prevents caspase 8 cleavage, but despite the lack of cleavage this drives apoptosis (Prakasam et al., 2014). The interaction between the different phospho-sites identified in these studies and the context specificity of this impact requires further elucidation. In addition to extrinsic apoptosis signalling, BID is a mediator of intrinsic apoptosis. Following DNA-damage during S-phase BID is phosphorylated at S61 by the DNA damage kinase ATM and functions to maintain the IR-induced cell cycle checkpoint (Kamer et al., 2005; Zinkel et al., 2005). In addition, increased BID S66 phosphorylation is required to induce apoptosis of cells stalled at the mitotic checkpoint, with a non-phosphorylatable form of BID suppressing cell death following mitotic arrest (Wang et al., 2014).

### BIM

BIM is a component of the intrinsic apoptosis cascade which competes for binding of pro-survival BCL-2 family members (Kim et al., 2006). There are multiple isoforms of BIM which all compete with BAX and BAK for binding to BCL-2 family proteins. Initially three isoforms were identified BIM_EL_, BIM_L_, and BIM_s_ (O’Connor et al., 1998). However, further work has identified a further isoform, BimAD, which shares capacity to induce apoptosis (Marani et al., 2002). The expression and stability of BIM is a key determinant of cell apoptotic potential, as demonstrated by the genetic requirement for BIM during MYC-induced apoptosis in multiple tissues (Muthalagu et al., 2014). Multiple target residues within BIM are phosphorylated to regulate stability. For example, in response to nerve growth factor (NGF), phosphorylation of BIM at S109 and T110 was detected in a MEK dependent manner, protecting neurons from BIM-induced cell death (Biswas and Greene, 2002). The phosphorylation of BIM by ERK1/2 at S65 and S69 following growth factor stimulation destabilising the protein and suppresses apoptosis (Ley et al., 2003; Luciano et al., 2003; Ley et al., 2004; Ley et al., 2005b). The activity of ERK mediated phosphorylation of BIM is also required to promote cell survival following detachment of cells (Fukazawa et al., 2004). Phosphorylation at S44, T56, and S58 by JNK drives apoptosis by activating BIM (Lei and Davis, 2003). A second study also found JNK mediated phosphorylation of BIM by JNK drives activation of mitochondrial driven mitosis in neurons. However, this study identified S65 as the target site (Putcha et al., 2003). The phosphorylation of BIM by both ERK and JNK suggests a complex interplay between the two kinases (Ley et al., 2005a). Finally, in a B cell line dependent upon interleukin-3 (IL-3) survival is determined by phosphorylation of BIM. In this cell line the phosphorylation of S87 by AKT is sufficient to drive ubiquitination and promote survival (Qi et al., 2006).

As with other members of the cell death induction pathway BIM has also been identified to have roles regulating mitosis dependent apoptosis. Aurora A has been identified to phosphorylate BIM at S93, S94, and S98 and drives degradation, this downregulates apoptotic signals through mitosis and supports successful completion of cell cycle (Moustafa-Kamal et al., 2013). The phosphorylation in a MEK dependent pathway was also required to successfully progress through mitosis (Graos et al., 2005).

### PUMA/NOXA

Both PUMA and NOXA are members of the BCL-2 family protein family that are expressed following p53 activation, for instance in response to DNA damage (Oda et al., 2000; Han et al., 2001; Nakano and Vousden, 2001; Yu et al., 2001). Both proteins are crucial mediators of p53 induced cell death (Villunger et al., 2003). The phosphorylation of NOXA by CDK5 at S13 is critical for suppressing its apoptotic function when glucose is available. This was confirmed by knockdown and ectopic expression of S12A NOXA mutants which increased apoptosis induction (Lowman et al., 2010; Karim et al., 2015). Interestingly PUMA interacts with HER2 and is phosphorylated at Y152 and Y172 this phosphorylation increased degradation of the protein and reduces apoptosis induction (Carpenter et al., 2013).

### BAX/BAK

BAX and BAK form pores in the outer mitochondrial membrane and drive mitochondrial permeabilization (Wei et al., 2001; Pena-Blanco and Garcia-Saez, 2018). While the activity of these proteins is primarily controlled by their release from pro-survival BCL-2 complexes, phosphorylation can also modify their activity at the mitochondrial membrane. The phosphorylation of BAX by GSK3β at S163 reduced both localisation to the mitochondria and pore formation, a finding which could be recapitulated by over-expression of a S163A BAX mutant (Linseman et al., 2004; Arokium et al., 2007). The initial mechanism identified for BAK phospho-regulation was regulation by non-receptor tyrosine phosphatases (PTPNs). The phosphate groups at Y108 and S117 must be removed to support the full activation of BAK apoptotic activity (Fox et al., 2010; Azad et al., 2012). More recently, the kinase responsible for the phosphorylation of these tyrosine residues has been identified as the protein kinase BMX (Fox and Storey, 2015).

### BCL-2 family

As previously discussed, the pro-survival proteins - BCL-2, BCL-XL, MCL-1, and BCL-W act to antagonise cell death induction by binding to BAX and BAK. The phosphorylation of BCL-2 by JNK at T56, S70, T74, and S87 provided a critical link between mitogenic signalling and propensity of cells to induce apoptosis (Maundrell et al., 1997). The importance of S87 for BCL-2 stability and activity was further demonstrated as mutation of S87A drives degradation of the protein (Breitschopf et al., 2000). P38 MAPK drives phosphorylation of the S87 and T56 sites (De Chiara et al., 2006). Finally, PKCα can phosphorylate BCL-2 S70 and this increases the resistance to apoptosis (Ruvolo et al., 1998). A study tried to reconcile the specific impact of each phosphorylation event upon the induction of apoptosis. The development of this model permitted prediction of apoptosis induction based on the modelling of BCL-2 phosphorylation status (Song et al., 2019). The phosphorylation of MCL-1 by ERK at T92 and T163 is required to prevent degradation of MCL-1 and thereby promote cell survival (Ding et al., 2008). The production of H_2_O_2_, is a carefully balanced cellular signalling event, and over-production can lead to significant cellular toxicity and apoptosis induction. The identification of JNK dependent phosphorylation of S121 and Thr163 of MCL-1 in conditions of high H_2_O_2_ was instrumental in linking high ROS to apoptosis induction (Inoshita et al., 2002). Interestingly, the phosphorylation of MCL-1 S159 and T163 is also controlled by dephosphorylation through PP2A (Slomp et al., 2021).

Phosphorylation events involved in mediating the function of pro- and anti-apoptotic proteins and thus cellular apoptosis are summarised in Table 2.

**TABLE 2:** Phosphorylation events regulating cellular apoptosis. BAD, BCL-2 associated agonist of cell death; BAX, BCL-2-associated X protein; BID, BH3 interacting-domain death agonist; BIM, BCL-2 interacting mediator of cell death; BCL-2, B cell lymphoma 2; CDK5, cyclin-dependent kinase 5; CKI, casein kinase I; CKII, casein kinase II; ERK, extracellular signal-regulated kinase; GSK3β, glycogen synthase kinase-3 beta; HER2, human epidermal growth factor receptor 2; JNK, Jun N-terminal kinase; MEK, mitogen-activated protein kinase kinase; MCL-1, myeloid cell leukaemia sequence 1; NOXA, phorbol-12-myristate-13-acetate-induced protein 1; PAK1, P21 (RAC1) Activated Kinase 1; PKCα, protein kinase C alpha; PUMA, p53 upregulated modulator of apoptosis.

Target	Phospho site	Kinase	Associated outcome
BAD	S112, S136	AKT	Suppression of apoptosis induction
BAD	S112 + S136 + S155	AKT	BAD degradation
BAD	S111	PAK1	Promotes cell survival
BID	S61, S64	CKI, CKII	Suppression of apoptosis induction
BID	T59	JNK	Apoptosis induction despite suppressed caspase-8 cleavage
BID	S61	ATM	Maintenance of the IR-induced cell cycle checkpoint
BID	S66 (increased phosphorylation)	Undefined	Induction of apoptosis following mitotic arrest
BIM	S109, T110	MEK	Neuron protection against BIM-induced cell death
BIM	S65, S69	ERK1/2	Suppression of apoptosis
BIM	S44, T56, S58	JNK	Activation of BIM to induce apoptosis
BIM	S65	JNK	Activation of mitochondrial driven mitosis in neurons
BIM	S87	AKT	Induction of ubiquitination to promote cell survival
BIM	S93, S94, S98	Aurora A	BIM degradation to promote successful completion of the cell cycle
NOXA	S13	CDK5	Suppression of apoptosis upon glucose availability
PUMA	Y152, Y172	HER2	PUMA degradation and suppression of apoptosis induction
BAX	S163	GSK3β	Reduction of BAX mitochondrial localisation and pore formation
BAX	Y108	BMX	Maintenance of BAX in an inactive conformation to prevent apoptosis
BCL-2	T56, S70, T74, S87	JNK	Induction of mitogenic signalling to promote apoptosis?
BCL-2	S70	PKCα	Increased resistance to apoptosis
MCL-1	T92, T163	ERK	Prevention of MCL-1 degradation to promote cell survival
MCL-1	S121, T163 (high H_2_O_2_ conditions)	JNK	Induction of ROS-driven apoptosis

### Modification of cell death regulators support disease development

The development of diseases, such as cancer, is often reliant upon the avoidance of apoptosis, with one key mechanism of disease adaptation involving the modulation of BH3 family expression levels (Certo et al., 2006). A second key adaptative mechanism in disease, is altered regulation of apoptotic protein post-translation modification. For example, the phosphorylation of BAD S118 and S99 has been linked to altered activity of mitochondrial electron transport chain utilisation, which supports tumour growth (Mann et al., 2019). Therefore, understanding the kinase pathways responsible for these changes provides novel treatment targets and elucidates treatment resistance mechanisms. The MAPK signalling pathway targets multiple sites which support the development of cancer. The overexpression of BRAF decreases anoikis induced cell death in melanoma cells through increased phosphorylation of BAD S75 (Boisvert-Adamo and Aplin, 2008). Additionally, the overexpression of B-RAF-V600E drove increased proliferation of cells in a BAD dependent manner (Polzien et al., 2011). In melanoma, BAD is phosphorylated through MEK/ERK dependent signalling, supporting cell survival, this is an adaptation from melanocytes where these phosphorylation’s are mainly driven by the RSK signalling pathway (Eisenmann et al., 2003). The adaptation of MAPK signalling also supports the development of blood cancers. A common mutation during the development of acute myeloid leukaemia (AML) is the FLT3-ITD mutation (Nakao et al., 1996; Griffith et al., 2004). In this setting, the activation of ERK/MEK pathway drives anti-apoptotic phosphorylation of BAD and suppresses cell death (Watanabe et al., 2019). Further, the overexpression of PIM3, a commonly observed oncogene in AML, utilises these mechanisms to suppress tumour cell death (Luo et al., 2020). The regulation of cell death proteins by PIM3 can also be observed in pancreatic cancer. Whereby, the PIM3 mediated phosphorylation of BAD is constitutive and is vital for prevention of apoptosis (Li et al., 2006). The stem cell niche is also supported by the phosphorylation of BIM in response to mitogenic signalling through the ERK pathway (Harada et al., 2004). Finally, the modulation of the MAPK family kinases in breast cancer has multiple impacts upon tumour survival. Over expression of HER2 is a common adaptation found in breast cancer cells. The direct phosphorylation of PUMA by HER2 provides a mechanism by which this initial adaptation can drive cell survival and suppression of apoptosis (Carpenter et al., 2013). MCL-1 phosphorylation by ERK is supported by PIN1 and supports survival of breast cancer cells through stabilisation of MCL-1 (Ding et al., 2008). The increased activity of the ERK signalling pathway in mammary epithelial cells also drives phosphorylation of BIM and prevents apoptosis induction and supports avoidance of anoikis which promotes metastasis (Marani et al., 2004; Collins et al., 2005). The overexpression of BAG-1 promotes phosphorylation at RAF driven BAD sites and inhibits apoptosis in breast cancer (Kizilboga et al., 2019).

The lipid secondary messenger kinase pathways support the adaptation of cell death pathways in cancer. For example, multiple members of the PKC family can drive phosphorylation of BAD. In prostate cancer cells PKCε can drive phosphorylation of S112 and promotes cell survival (Meshki et al., 2010). In parallel, G-protein coupled oestrogen receptor 1 (GPER), can drive increased activity of PKA signalling which increases BAD phosphorylation and increases breast cancer cell survival (Chan et al., 2020). In both Glioma and lung cancer, PKC iota phosphorylates S112 and promotes cell survival (Jin et al., 2005; Desai et al., 2011). BAD expressed in neuronal cells can be phosphorylated by PINK1 at S112 and can promote survival in these cell types (Wan et al., 2018). Increased AKT activation and phosphorylation of BAD is associated with the adaptation of the breast cancer cell line MCF-7Ca cells to oestrogen deprivation (Sabnis et al., 2005).

The interplay between these pathways has been assessed in melanocytes, suggesting inhibition of adapted ERK/PKA/PKC pathways simultaneously yields the highest apoptosis induction (Sastry et al., 2020). This phenotype can also be seen in immortalised epidermal keratinocytes which are dependent upon BIM to prevent anoikis (Quadros et al., 2006).

The DNA damage kinase cascade has also been demonstrated to support modification of cell death related proteins. The phosphorylation of BID by ATM is critical for supporting the hematopoietic stem cell niche and is therefore a prime target for kinase driven adaptation in disease development (Maryanovich et al., 2012). This is supported by the increased development time to leukemogenesis in ATM −/− mice is BID expression is also suppressed, suggesting BID is required to prevent apoptosis of leukemic cells (Biswas et al., 2013).

This underlying adaptation of kinase signalling in tumours can have impacts beyond tumour initiation. This can also define the resistance to chemotherapeutic intervention. For instance, MAPK pathway activation can induce BIM dependent resistance to paclitaxel in epithelial tumours (Tan et al., 2005). The phosphorylation of BAK by BMX increases the resistance of cancer cells to chemotherapy, suggesting this adaptation supports the disease persistence (Fox and Storey, 2015). The development of resistance to tyrosine kinase inhibitors is a typical adaptation in treated non-small cell lung cancer. This is mechanistically supported by the phosphorylation of MCL-1 which stabilises the protein and increases expression levels (Wu et al., 2016). Resistance of ovarian cancer to chemotherapies can be closely tied to phosphorylation of BAD (Marchion et al., 2011). The phosphorylation of key residues in the apoptotic proteins is modulated by a balance between kinases and phosphatases. Therefore, it is unsurprising that modulation of PP2A activity regulates the dephosphorylation of S112 and mediates the sensitivity of chronic lymphocytic leukaemia (CLL) cells to cAMP pathway inhibition (Moon and Lerner, 2003).

### Targeting cellular apoptosis in disease

Cellular apoptosis has a dual role in disease, with cancers usually resisting cell death by apoptosis and neuronal apoptosis implicated in various neurological diseases. BAD phosphorylation is perhaps the most characterised apoptosis-related phosphorylation event that has demonstrated promise in the cancer therapeutic landscape. Treatment with the PI3K inhibitor Piticlisib (GDC-0941) leads to BAD dephosphorylation at S112 and S136 sensitising glioblastoma cells to the BCL-2 inhibitor ABT-263 (Pareja et al., 2014). A study investigating novel treatments for peritoneal carcinomatosis, discovered that a combination of mitomycin C (a DNA crosslinker) and the mTOR inhibitor rapamycin successfully induced apoptosis by BAD dephosphorylation by inactivating p70 S6 ribosomal kinase (S6K1) (Song et al., 2014). These examples serve as proof of concept that targeting phosphorylation events implicated in apoptosis could be a promising anti-cancer approach. Additionally, combination therapies as discussed in the context of Debio 1143 + PD-1/PD-L1 checkpoint blockade and Piticlisib + ABT-263 suggest a possibility for kinase targeting to serve as a sensitisation strategy rather than a single-agent therapeutic in anti-cancer regimens. Dexmedetomidine (DEX) is a commonly used sedation medication that exerts its action via agonism of the central pre- and postsynaptic α2-receptors in the locus coeruleus (Weerink et al., 2017). Interestingly, preclinical efforts have shown that DEX could reduce brain damage after ischemia-reperfusion by decreasing ER stress-induced neuronal apoptosis (Zhai et al., 2019). DEX treatment also reduced phosphorylated-JNK suggesting in this context JNK may have pro-apoptotic properties considering its role of a double-edged sword in apoptosis regulation. This evidence of kinase activity modulation as a downstream effect of pharmacological interventions mediating cellular apoptosis highlights the promise behind direct kinase targeting which is currently a considerably under-researched area. DEX’s translational potential was investigated in an interventional clinical trial hoping to show neuroprotective effects of DEX administration during brain surgery by means of reduced apoptosis and subsequent neuronal injury (NCT02878707).

Current clinical attempts in the cancer therapeutic landscape aim to induce apoptosis in a selective manner through multiple mechanisms. Debio 1143 (AT-406, SM-406) is a second mitochondrial-derived activator of caspases (SMAC) mimetic and acts as antagonist of the inhibitor of apoptosis proteins (IAPs) aiming to restore apoptosis in cancer cells (Cai et al., 2011). A different approach to inducing apoptosis is the inhibition of BCL-2 to prevent it from opposing pro-apoptotic protein function. A phase I clinical trial is investigating the BCL-2 inhibitor TQB3909 for the treatment of patients with relapsed or refractory advanced malignant tumours (NCT04975204). The variety of pharmacological efforts to restore apoptosis in cancer cells confirms the need to explore diverse upstream of apoptosis signalling events (incl. phosphorylation) to combat the heterogenous nature of cancer.

## Regulation of mitochondrial calcium homeostasis—channel activity and mitochondrial associated membranes

**FIGURE 7:**
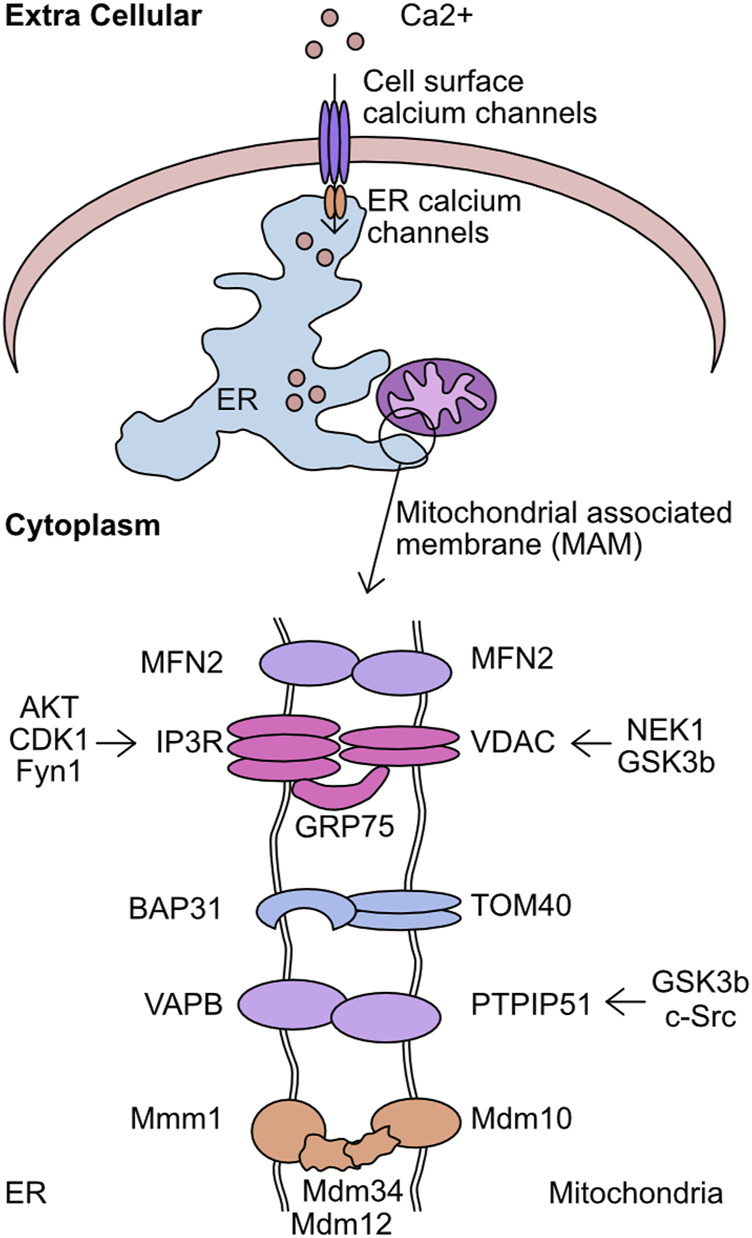
Overview of kinases which target the mitochondrial associated membrane (MAM). The mitochondrial associated membrane (MAM) is the association point between mitochondria and endoplasmic reticulum (ER). This complex is stabilised by the activity of 5 complexes–MFN2/MFN2, IP3R/GRP75/VDAC, BAP31/TOM40, VAPB/PTPIP51, and Mmm1/Mdm12/Mdm34/Mdm10. This complex is critical for mediating calcium transfer from extracellular sources, through the ER, and finally into the mitochondria. Shown are the components of the MAM complexes and the identified kinases which target each member.

The level of cellular Ca^2+^ exists in a carefully regulated equilibrium. The proteins involved in detecting cellular calcium levels, mediating its entry through the plasma membrane, and facilitating intra-cellular transfer, are critical to correct cellular function. Movement of calcium from the extracellular space into the ER is regulated by store operated calcium entry (SOCE) (Putney, 2011). In short, this mechanism acts to monitor ER calcium levels and upon depletion plasma membrane channels are activated to refill these depleted stores. The correct functioning of these systems prevents the pathological effects of Ca^2+^ depletion or equally detrimental Ca^2+^ overload. Once stored in the ER, Ca^2+^ can be mobilised by stimulation of ER receptor channels, namely, the IP_3_ receptor (IP3R) and ryanodine receptors (RYR) (Streb et al., 1983; Otsu et al., 1990). In particular, the interaction sites between the ER and mitochondria are key intracellular transfer hubs for Ca^2+^ (Csordas et al., 2006; Csordas et al., 2010). These sites are referred to as mitochondria associated membranes (MAM) or Mitochondrial-ER contact sites (MERCs) and are defined by the <20 nm distance between the ER/mitochondrial membranes and distinguishing protein composition (Vance, 1990; Rizzuto et al., 1998). The membranes are stabilised by the formation of multiple structural complexes: IP3R/GRP75/VDAC1, dimers of MFN2, VAPB/PTPIP51, TOM40/BAP31, and the Endoplasmic Reticulum-Mitochondria Encounter Structure (ERMES) (Figure 7) (Szabadkai et al., 2006; de Brito and Scorrano, 2008; Kornmann et al., 2009; De Vos et al., 2012; Namba, 2019). The maintenance of these interaction sites has multiple implications for mitochondrial function and morphology. The loss of mTORC1 signalling drives altered MERC structure which reduces mitochondrial cristae formation in an MFN2 dependent manner, directly altering metabolic capacity (Sood et al., 2014). MAM promotes DRP1 mediated mitochondrial fission by constricting the mitochondrial membrane prior to recruitment of DRP (Friedman et al., 2011). These sites also play a critical function as lipid exchange sites between the mitochondria and ER. Enzymes responsible for the synthesis of phosphatidylserine (PS), phosphatidylethanolamine (PE), and phosphatidylcholine (PC) are enriched at these contact sites (Vance, 1991). However, the complexities of this process and the regulatory systems is still an expanding area of research. Given these functions, it is unsurprising that these sites are linked to metabolic diseases (Theurey and Rieusset, 2017), and the loss of MAM association can increase glycolysis utilisation and boost cancer cell resistance to chemotherapeutics (Cardenas et al., 2016; Madreiter-Sokolowski et al., 2016).

The entry of Ca^2+^ into the mitochondrial matrix is mediated by outer membrane localised voltage dependent anion channels (VDAC). There are three VDACs in the human genome, VDAC1, VDAC2, and VDAC3, with these being the predominant route through which calcium transitions the outer mitochondrial membrane (Gincel et al., 2001; Rapizzi et al., 2002; De Stefani et al., 2012). Once across the mitochondrial outer membrane the mitochondrial uniporter (MCU) facilitates calcium flux from the intermembrane space into the mitochondrial matrix. The discovery of this critical mitochondrial calcium regulator has been previously reviewed in depth (Raffaello et al., 2012). Once in the mitochondrial matrix calcium has multiple roles including as a co-factor for enzymes, such as pyruvate dehydrogenase phosphatase 1 (PDP1) (Karpova et al., 2003). Upon binding to calcium PDP1 dephosphorylates S293 of pyruvate dehydrogenase (PDH). The removal of this phosphorylation increases PDH enzymatic activity both *in vivo* and *in vitro* and provides a link between mitochondrial calcium homeostasis and metabolism (Mallilankaraman et al., 2012; Pan et al., 2013). This increased activity of PDH was hypothesised to impact ATP production in mitochondria. However, conflicting evidence has been generated in this regard. While the KO of MCU in a murine model increased p-PDH levels in samples derived from skeletal muscle, mouse embryonic fibroblasts (MEFs) derived from this model had normal basal respiratory rate, despite reduced mitochondrial calcium. Interestingly, MCU KO mice had a reduced capacity to cope with strenuous physical activity, a process closely associated cellular energy production capacity (Pan et al., 2013). This is in contrast to the loss of MCU in pancreatic β-cells reducing ATP production when stimulated with glucose and histamine mediated mitochondrial calcium flux increasing cytosolic and mitochondrial ATP levels in HeLa cells (Jouaville et al., 1999; Alam et al., 2012). Importantly, these studies investigated the impact of MCU loss in very different cellular contexts. It is conceivable that MCU mediated mitochondrial calcium influx is more closely associated with basal respiration in specific cell types. Additionally, the dependence of basal respiration upon MCU mediated calcium influx may change depending on the level of cellular energy stress. Therefore, in cancer cell lines or during intense physiological activity.

In addition to this metabolic role, mitochondrial calcium homeostasis can have significant impact upon the capacity of cells to induce apoptosis. For example, the increased flux of calcium between the endoplasmic reticulum (ER) and mitochondria by IP_3_ mediated release following ceramide treatment supports apoptosis induction (Szalai et al., 1999; Csordas et al., 2002). In addition, ceramide was found to induce flux of calcium from the ER into the mitochondrial matrix, inducing gross morphological changes (Pinton et al., 2001). However, in another study, increased expression of BCL-2 increased both mitochondrial calcium and membrane potential while rendering the cells more resistant to apoptotic stimuli, with the block of calcium uptake re-sensitising cells to apoptosis (Zhu et al., 1999). This may be linked to the capacity of DRP1 mediated mitochondrial network fragmentation to prevent calcium overload and protect cells from specific modalities of apoptosis inducer (Szabadkai et al., 2004).

The capacity of these processes to drive altered metabolism and modulate sensitivity to apoptosis makes them key targets to support persistence of disease states. While protein phosphorylation has been linked to regulation of both mitochondrial calcium regulation and MAM development, the full extent to which these processes contribute is still poorly understood.

### Mitochondrial calcium homeostasis

One of the first reports of phosphorylation mediated regulation of mitochondrial calcium overexpressed multiple PKC isoforms. Each PKC isoform had a differential impact upon cellular calcium stores, with PKCζ increasing histamine mediated calcium flux into mitochondria (Pinton et al., 2004). While no phospho-target was determined for this activity it suggested the importance of post-translational modification for mitochondrial calcium regulation. VDAC complexes form pores on the outer mitochondrial membrane responsible for the uptake of Ca^2+^. In addition to this role, some reports have implicated a role for VDAC in MOMP (Kim et al., 2019). Importantly, the phosphorylation of VDAC can both inhibit and promote MOMP. While the phosphorylation of VDAC1 by NEK1 at S193 protects cells from induction of mitochondrial membrane permeabilization (Chen et al., 2009; Chen et al., 2010). The phosphorylation of VDAC1 by GSK3β at S12 and S103 increases sensitivity to endostatin induced death by increasing VDAC1 expression levels and supporting mPTP opening and caspase 3 activation (Yuan et al., 2008). The mechanisms by which phosphorylation mediates this effect is incompletely understood. However, one key element is loss of hexokinase II (HK2) binding to VDAC1, which can act to protect against cell death in response to chemotherapy in cancer cells (Pastorino et al., 2005). It is also possible that the effect is mediated by the effectiveness of MCU to permit mitochondrial calcium entry. The identification of a tyrosine phosphorylation event on MCU driven by PYK2 activity resulting in MCU oligomerisation suggests an acute phosphorylation-driven method of regulating mitochondrial calcium (O-Uchi et al., 2014). This is further supported by the AMPK mediated phosphorylation of MCU, which drives a mitotic specific calcium influx to drive increased ATP production, with loss of this regulation leading to spindle assembly delay (Zhao et al., 2019). Finally, PKC mediates phosphorylation of S92, while not functionally characterised, is hypothesised to alter the dimerization capacity of MCU tetramers (Lee et al., 2020). This functional activation of MCU is predominantly regulated by the complex subunits MICU1 and MICU2 (Kamer et al., 2017; Wang et al., 2020). The phosphorylation of MICU1 by AKT at S124 increases mitochondrial calcium (Marchi et al., 2019). The NA(+)/Ca(2+) exchanger (NCLX) is a major route of calcium efflux from the mitochondria into the cytoplasm (Palty et al., 2010). The phosphorylation of NCLX by PKA can rescue the typical calcium overload observed in PINK1 deficient cells, this mechanism is critical for maintenance of neuronal cell viability (Kostic et al., 2015; Rozenfeld et al., 2022).

### Mitochondrial associated membrane (MAM) regulation

A major finding was the identification of a promyelocytic leukaemia (PML) body at MAMs, which associated with IP3R, AKT, and PP2A. Furthermore, this AKT/PP2A mediated regulation of IP3R was critical to the regulation of calcium release (Zaccolo and Pozzan, 2002). This supported previous work which suggested the phosphorylation of IP3R by AKT at S2681 reduced the efflux of calcium from the ER and protected cells from apoptosis (Marchi et al., 2008; Szado et al., 2008). AKT is also the kinase responsible for phosphorylation of Phosphofurin acidic cluster sorting protein 2 (PACS-2) at S437, which increases its association with 14-3-3 proteins preventing its role in apoptosis. This association can be reversed by the addition of TRAIL to cells which induces dephosphorylation and promotes apoptosis (Aslan et al., 2009). This cascade was further elucidated by the finding that MTORC2 is responsible for the activation of AKT in these conditions (Betz et al., 2013). In addition to these phosphorylation dynamics during apoptosis regulation, the control of these processes has been observed in a cell cycle dependent manner. The modification of IP3R1 by CDK1/cyclin B complex at T799 drives increased IP3 binding and Ca^2+^ release, suggesting a cell cycle dependency (Malathi et al., 2005). Finally, altered calcium signalling is a key element of cellular differentiation. The phosphorylation of IP3R Y353 by Fyn kinase drives prolonged calcium release from the ER during B cell activation, linking this protein with developmental Ca^2+^ release (Cui et al., 2004; deSouza et al., 2007).

The modification of MAM structural proteins is not limited to IP3R and VDAC components. The interaction between VAPB-PTPIP51 is impacted by GSK3β with the inhibition of kinase activity increasing the complex formation (Stoica et al., 2014). In addition, PTPIP51 was also found to be phosphorylated at T176 by c-Src. The altered phosphorylation of this site drove differential interaction with 14-3-3 family proteins and PKA (Brobeil et al., 2012). Given this interaction with 14-3-3 proteins, this could impact cellular localisation of PTPIP51. The impact of this phosphorylation site upon the activity of PTPIP51 in MAM control has not been investigated.

Evidently both Ca^2+^ homeostasis and MAMs are tightly regulated by phosphorylation with all discussed phosphorylation events summarised in Table 3.

**TABLE 3:** Phosphorylation events regulating mitochondrial calcium homeostasis and MAMs. CDK1, cyclin-dependent kinase 1; GSK3β, glycogen synthase-3 beta; IP3R1 inositol 1,4,5–triphosphate receptor type 1; MCU, mitochondrial calcium uniporter; MICU1, mitochondrial calcium uptake 1; NEK1, NIMA-related kinase 1; PACS-2, phosphofurin acidic cluster sorting protein 2; PTPIP51, protein tyrosine phosphatase interacting protein 51; VDAC1, voltage dependent anion channel type 1.

Target	Phospho site	Kinase	Associated outcome
VDAC1	S193	NEK1	Suppression of mitochondrial membrane permeabilisation
VDAC1	S12, S103	GSK3β	Increased sensitivity to endostatin-induced cell death
MCU	S92	PKC	Altered dimerization capacity of MCU tetramers (hypothesis)
MICU1	S124	AKT	Increase of mitochondrial Ca^2+^
PACS-2	S437	AKT	Prevention of PACS-2’s role in apoptosis
IP3R1	T799	CDK1/cyclin B	Increase in IP3 binding and Ca^2+^ release
PTPIP51	T126	c-Src	Support of correct interaction with 14-3-3 family proteins and PKA

### Modification of MAM proteins and mitochondrial transporters supports disease development

The importance of mitochondrial Ca^2+^ regulation and MAMs makes them common points of adaptation in disease. While mitochondrial lipid homeostasis, mitochondrial Ca^2+^ and metabolism are regulated by MAM proteins, the investigation of how these pathways drive disease is still developing. Some promising work suggests a role for regulation of these processes by kinase signalling. One study has shown interaction of RIPK1 with MCU increases mitochondrial Ca^2+^ level and promotes proliferation of colorectal cancer cells (Zeng et al., 2018). The importance of Ca^2+^ in cancer is further supported by the AKT mediated phosphorylation of MICU1 S124 promoting proliferation of three different oncogenic cell lines (Marchi et al., 2019). Finally, the dysregulation of mitochondrial function is increasingly associated with the onset of neurological diseases. The regulation of VAPB-PTPIP51 in amyotrophic lateral sclerosis and frontotemporal dementia (ALS/FTD) by FUS metabolism defects through GSK3β mediated regulation of complex formation and supports disease development (Stoica et al., 2016).

### Targeting mitochondrial Ca^2+^ homeostasis and MAMs in disease

There is an intricate relationship between MAMs and mitochondrial Ca^2+^ homeostasis. Hence it is not surprising that therapeutic approaches aimed at stabilising the mitochondria-ER assembly have demonstrated promise in the treatment of neurological diseases driven by defective MAMs and dysregulated Ca^2+^ signalling. While current clinical trials have not targeted the kinases which regulate these proteins, the ongoing studies show the potential promise of such an approach.

The Sigma-1 receptor (Sig-1R) is a chaperone at the ER (in MAMs) that maintains Ca^2+^ homeostasis and MAMs stability among others. Sig-1R is sensitive to Ca^2+^ concentrations and mediates Ca^2+^ signalling through IP3R. Sig-1R deficiency or absence negatively impacts mitochondria-ER contacts and Ca^2+^ homeostasis inducing neuron degeneration and other symptoms of neurological/neurodegenerative disorders. Therefore, Sig-1R agonists including Pridopidine (ACR16) presented as a promising therapeutic opportunity. A phase 3, randomised, double-blind, placebo-controlled trial investigated the effect of Pridopidine in Huntington’s disease (HD) patients (de Yebenes et al., 2011) (NCT00665223, MermaiHD). While no change in the modified motor score was observed at 26 weeks, the authors suggested that Pridopidine may influence the motor phenotype in HD. A phase 2/3 study, (NCT04615923), part of HEALEY ALS (NCT04297683), investigated the use of Pridopidine as a potential treatment of amyotrophic lateral sclerosis (ALS). While the study did not meet its primary endpoint at 24 weeks–positive change in ALS Functional Rating Score-Revised (ALSFRS-R), ALS patients on Pridopidine presented with improved speech measures.

There is evidence of successful preclinical targeting of MCU to inhibit Ca^2+^ exchange between the ER and mitochondria in models of Parkinson’s disease (Soman et al., 2017; Liu et al., 2020). Additionally, the mitochondrial MAM protein cyclophilin D is vital for MAM integrity and interactions required for efficient insulin signalling (Tubbs et al., 2014). These data suggested modulating MAMs and mitochondrial Ca^2+^ homeostasis to be a viable therapeutic strategy. Further work is required to investigate the potential of kinase targeting in this context.

## Reactive oxygen species (ROS) a critical kinase regulated mitochondrial pathway

A by-product of energy generation through the electron transport chain are reactive oxygen species (ROS). ROS can be separated into two groups: radical and non-radical oxygen species. The radicals constitute superoxide (O_2_^•-^) and hydroxyl radicals (HO^•^), these molecules are highly reactive due to an unpaired electron and capable of modifying cysteine residues within proteins and oxidising lipid membranes. Conversely, hydrogen peroxide (H_2_O_2_) is non-radical and is membrane permeable. However, while not initially reactive this molecule is stable and can decompose into hydroxyl radical by multiple mechanisms, including reaction with metal groups such as Iron through the Fenton reaction (Sabharwal and Schumacker, 2014). To protect against the activity of these radicals, multiple protein families have evolved to support detoxification of ROS. To prevent the conversion of O_2_^•-^ into the more toxic HO^•^ radical the superoxide dismutase (SOD) family instead converts the initial O_2_^•-^ into H_2_O_2_. There are three members of this family known as SOD1, SOD2, and SOD3, which are transcribed from different genes but also utilise different metal catalyst ions at their active site. A comprehensive review of these proteins (Perry et al., 2010). Once formed, H_2_O_2_ oxidises glutathione (GSH) through the activity of one of 8 human glutathione peroxidases (GPx) to form glutathione disulfide (GSSG) and a water molecule. The GSSG is then resolved back to GSH by the activity of thioredoxin (Holmström and Finkel, 2014; Brigelius-Flohe and Flohe, 2020).

The modulation of cellular ROS levels is critical as this molecule is a crucial signalling molecule at tolerable levels but a cellular toxin when produced in excess. Phosphatases in particular are highly sensitive to oxidation by H_2_O_2_. For example, oxidation can reversibly inactivate PTEN and elevate AKT signalling, promoting proliferation (Lee et al., 2002). However, uncontrolled ROS can react with Fe-clusters and induce extensive lipid peroxidation leading to ferroptosis (Dixon et al., 2012; Stockwell et al., 2017; Hassannia et al., 2019). Many of the proteins in these response cascades are modulated by cysteine oxidation. However, phosphorylation plays an essential role in defining activation of key components of mitochondria to nuclear retrograde signalling processes.

### Modulation of mitochondrial driven transcriptional response to ROS

**FIGURE 8:**
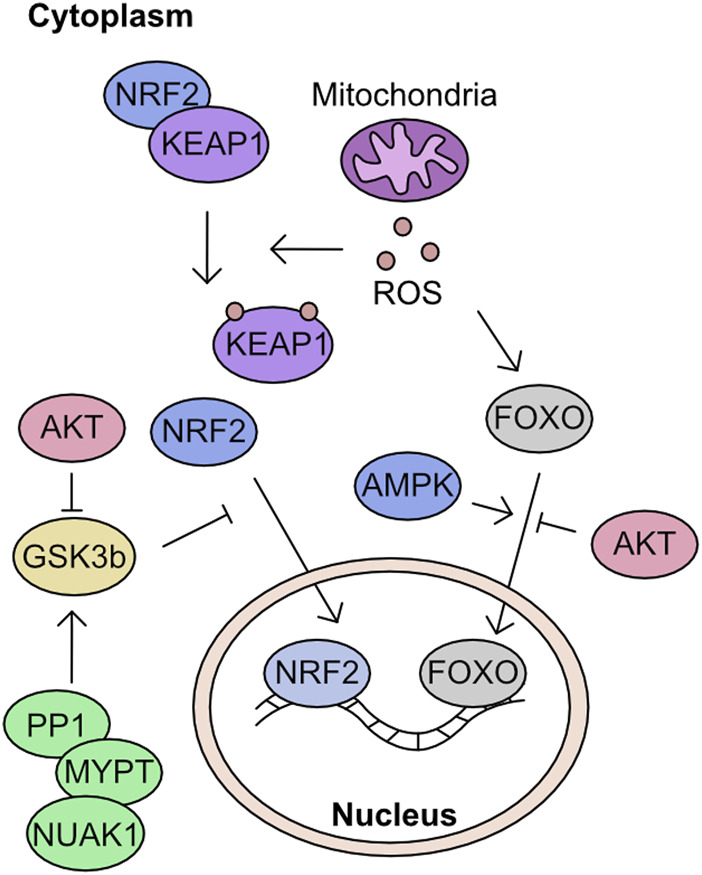
Overview of kinases which target Reactive oxygen species (ROS) response network. Mitochondria are a key cellular source of ROS. The generation of ROS is monitored by the KEAP1 cascade and fork head (FOXO) transcription factors. In situations of increased ROS, cysteines within KEAP1 are oxidised inducing the dissociation from NRF2. This unbound NRF2 is then prevented from entering the nucleus by a GSK3b mediated phosphorylation. The activity of GSK3b can be inhibited by AKT or through reduced activity of the PP1/MYPT/NUAK1 complex. The combined loss of phosphorylation and dissociation from NRF2 permits nuclear translocation and induction of transcriptional response. In parallel, ROS levels can trigger nuclear translocation of FOXO. This process can be inhibited by AKT mediated phosphorylation or promoted by AMPK.

The cellular response to high ROS levels partly mediated by transcriptional response which increase expression of detoxifying enzymes. The Nuclear respiratory factor (NRF) family consists of two members, NRF1 and NRF2. Both proteins are key regulators of nuclear localised mitochondrial genes. In particular, the oxidation of the cysteines in nuclear factor-erythroid factor 2-related factor 2 (NRF2) binding partner Kelch-like ECH-associated protein 1 (KEAP1), induces NRF2 dissociation and permits nuclear translocation activating multiple signalling cascades that support the cellular ROS response (Itoh et al., 1999; Sekhar et al., 2002; McMahon et al., 2003; Stewart et al., 2003). In addition to this dissociation with KEAP1, NRF2 nuclear translocation is also regulated by phosphorylation. GSK3β phosphorylates NRF2, preventing nuclear accumulation of the latter and this activity is countered by inhibitory phosphorylation of GSK3β on Ser9 by AKT (Table 4) (Rojo et al., 2008). Further complexity to this cascade emerged with the discovery that the AMPK-related kinase NUAK1 limits the rate of myosin phosphatase targeting subunit 1 (MYPT1)/PP1-dependent dephosphorylation of GSK3β Ser9 (Figure 8) (Port et al., 2018). The phosphorylation of S445, S472, and S910 by NUAK1 increases association of MYPT1 with PP1, inhibiting phosphatase activity. This prevents the dephosphorylation of GSK3β S9 and inhibits kinase activity. This prevents the dephosphorylation of GSK3β S9 and inhibits kinase activity. The suppression of GSK3β reduces the phosphorylation of NRF2 at canonical sites in the phospho-degron, S334 and S338, decreasing association with SCF/β-TrCP and subsequent degradation (Rada et al., 2011). This increases the pool of NRF2 available to induce signalling responses. Interestingly, in addition to the roles in redox control, NRF2 association with PGAM5 and KEAP1 was found to regulate mitochondrial motility, with the loss of either NRF2 or PGAM5 from this complex driving MIRO1 degradation and mitochondrial clustering (O'Mealey et al., 2017).

**TABLE 4:** Phosphorylation events regulating mitochondrial ROS. FOXO3a, forkhead factor of the O class 3a; GSK3β, glycogen synthase kinase-3 beta; MST1, Ste20-like kinases; NRF2, nuclear factor-erythroid factor 2-related factor 2; UCP2, Uncoupling protein 2; PGC-1α, Peroxisome proliferator-activated receptor gamma coactivator 1-alpha.

Target	Phospho site	Kinase	Associated outcome
NRF2	S334, S338	GSK3β	Inhibition of NRF2 nuclear import
GSK3β	S9	AKT	Suppression of GSK3β-driven inhibition of NRF2 nuclear import
FOXO3a	S207	MST1	Induction of nuclear translocation and subsequent cell death
FOXO3	S413, S588	AMPK	Increased transcriptional activity and subsequent expression of UCP2 and PGC1alpha
FOXO1	S22	AMPK	Decreased binding to 14-3-3 proteins
FOXO1	S383, T649	AMPK	Increased transcriptional activity

A second crucial ROS response pathway are the forkhead factors of the O class (FOXOs). All members of the FOXO family have conserved target sites which, when phosphorylated by AKT, increase association with 14-3-3 family members and traffic these proteins out of the nucleus. In proliferating cells this activity of AKT inhibits nuclear translocation of FOXO factors and prevents quiescence induction (Brunet et al., 1999; Kops et al., 1999; Rena et al., 1999). Given the importance of AKT for cellular ROS response multiple mechanisms have evolved to enable FOXO activity in the presence of increased AKT kinase activity. For example, a key mechanism of regulation is the deacetylation of FOXO3 by SIRT1 in conditions of oxidative stress increasing the expression of FOXO target genes (Brunet et al., 2004). In addition, multiple kinase pathways can promote increased activity of FOXO transcriptional activity. A kinase particularly associated with both mitochondrial function and ROS is AMPK, which plays a key role in control of FOXO factors. The AMPK-mediated phosphorylation of S22 decreases binding of FOXO1 to 14-3-3 proteins, increasing nuclear localisation, while the phosphorylation of S383 and T649 increase transcription of FOXO1 target genes, such as, mSOD (Yun et al., 2014; Saline et al., 2019). During nutrient deprivation, AMPK phosphorylates FOXO3 at six sites, but of note are S413 and S588. The presence of phosphorylation at these sites increases transcriptional activity of FOXO3 and drives expression of mitochondrial genes, such as, uncoupling protein 2 (UCP2) and PPARG coactivator 1 α (PGC1α) (Greer et al., 2007). This can also directly drive oxidative stress induced expression of SODs in quiescent cells and protect against apoptosis induction (Kops et al., 2002). Importantly the cross talk between transcriptional pathways can have significant impact of ROS response outcome. The activation of FOXO3a cascade can repress MYC-driven mitochondrial gene transcriptional programs, reducing mitochondrial function, and suppressing ROS production (Delpuech et al., 2007; Ferber et al., 2012). These mechanisms suppress apoptosis in response to oxidative stress. In contrast, the phosphorylation of FOXO3 at S207 by oxidation activated mammalian Ste20-like kinases (MST1) suppresses association with 14-3-3 proteins and promotes nuclear translocation, inducing cell death (Table 4) (Lehtinen et al., 2006).

### Modification of ROS transcriptional responses supports disease

The onset of disease is associated with altered cellular response to ROS. Tumours driven by MYC, KRAS^G12D^, and BRAF^V600E^ all show increased basal NRF2 transcriptional activity promoting tumour survival (DeNicola et al., 2011). The altered regulation of NRF2 regulation has been observed through multiple mechanisms. In BRCA1 mutant breast cancer overactive Pi3K/AKT signalling in releases GSK3β mediated suppression of NRF2 nuclear translocation, supporting tumour survival (Gorrini et al., 2014), while in colorectal cancer, the regulation of GSK3β activity by the NUAK1/MYPT/PP1 complex maintains nuclear localisation of nuclear NRF2 (Port et al., 2018). In addition, common loss of KEAP1 function in non-small cell lung cancer promotes over activity of NRF2 transcriptional cascades (Hast et al., 2014; Berger et al., 2016). In addition to supporting tumour cell survival this adapted ROS response can lead to treatment resistance (Scalera et al., 2022).

In addition to cancer, the dysregulation of ROS regulatory cascades is strongly associated with development and progression of neurodegenerative diseases (Barnham et al., 2004; Singh et al., 2019). The lipid membranes of neuronal cells are rich in poly unsaturated fatty acids (PUFAs). This lipid species is particularly sensitive to oxidative damage. The accumulation of α-synuclein (α-syn) increases ROS generation and damages lipid membranes leading to ferroptosis and neuronal cell death, with this mechanism contributing to the onset of Parkinson’s disease (Angelova et al., 2015). In a murine model of Alzheimer’s disease, increased lipid peroxidation was found to correlate strongly with subsequent deposition of amyloid β plaques, suggesting a contribution of oxidation to progression of the disease (Praticò et al., 2001).

### Therapeutic strategies mediating the cellular anti-oxidant response

The combination of elevated ROS and decreased endogenous antioxidants in cancer, neurodegenerative and cardiovascular diseases drove clinical and preclinical efforts to mitigate these phenotypes.

MIT-001 is a ROS scavenger that mediates graft-versus host disease via decreased high-mobility group box 1 (HMGB1) expression–an inflammatory marker and lowered oxidative stress (Im et al., 2015). An active phase II clinical trial is investigating the efficacy of three different MIT-001 doses as a preventative for oral mucositis (OM) in patients with head and neck squamous cell carcinoma HNSCC who are undergoing chemoradiotherapy (NCT04651634). Chemoradiotherapy-associated OM is usually caused by injury to basal epithelial cells inducing increased ROS levels. MIT-001 is expected to decrease ROS levels and excessive inflammation caused by the high ROS thus preventing the development of OM.

Nicotinamide riboside (NR)—a dietary supplement, is a nicotinamide adenine dinucleotide (NAD+) precursor showing promising therapeutic action via elevating the levels of NAD+ in the body (Bieganowski and Brenner, 2004). NAD+ is a coenzyme essential for numerous oxidation-reduction reactions in cells. Various NAD+ -depleting enzymes are known to be activated by oxidative stress thus exogenously elevating NAD+ levels presented as a promising antioxidant strategy in disease (Zhang et al., 2019). Several preclinical efforts supported this showing that NAD+ enhancing strategies improved outcomes in metabolic disorders (Cantó et al., 2012), neurodegenerative diseases (Schöndorf et al., 2018) and age-associated complications (Mills et al., 2016). An early phase I clinical trial is underway investigating whether NR supplementation can improve cardiac function in patients with heart failure (HF) (NCT04528004). A focus of the study will be to determine how increased blood and myocardial NAD+ levels affect mitochondrial function in HR.

Mitoquinone (MitoQ) comprises of Triphenylalkylphosphonium ion (TPP+) conjugated to the endogenous antioxidant Ubiquinone and is one of the best described mitochondria-targeted antioxidants (Kelso et al., 2001). MitoQ has been shown to reduce oxidative damage and display anti-apoptotic properties in various disease models further reiterating the complex relationship between mitochondria-driven processes (Duarte et al., 2023). MitoQ supplementation was investigated as a prevention strategy in breast cancer patients undergoing treatment with doxorubicin-adjuvant chemotherapy (NCT05146843). An expected outcome was reduction in doxorubicin-induced oxidative stress.

Interestingly, mediating oxidative stress is perhaps one of a few disease presentations with completed or ongoing clinical trials investigating dietary supplements (both NR and MitoQ among others) as the therapeutic strategy.

These studies in combination with the mechanistic evidence that GSK3β phosphorylation supports the antioxidant response machinery in cells suggest that targeting phosphorylation events associated with ROS decrease or antioxidant increase could be a viable therapeutic strategy.

## Conclusion and future directions

This review has covered the myriad of impacts protein phosphorylation can have on mitochondrial homeostasis. In particular, processes which control network morphology, apoptosis, calcium control, MAMs, and finally retrograde ROS signalling. While the regulation of these processes is not limited to only phosphorylation, this modification is key for defining protein function and the overall pathway response, especially in the context of perturbed kinase signalling in disease.

The understanding of these fundamental mitochondrial processes has already yielded projects to develop novel therapeutic strategies. Targeting aberrant mitochondrial morphology is a focus in cardio-protection and neurological diseases. Pharmacological modulation of apoptosis induction has proven to be a viable anti-cancer strategy with multiple clinical trials underway. On the other hand, neurological diseases induced by neuronal cell death are likely to benefit from anti-apoptotic strategies. Maintaining healthy mitochondrial Ca^2+^ homeostasis and supporting MAMs integrity presented as an approach to support complications in neurological disorders. Finally, combating high ROS levels by ROS scavengers or antioxidants is also under consideration in the clinic for multiple disease settings. In general, there have been considerable preclinical and clinical efforts to target the above-described aspects of mitochondrial biology confirming their importance and therapeutic potential (Table 5). However, a potential limitation of targeting mitochondrial associated phenotypes is the constitutive nature of these processes. Therefore, development of novel therapeutic interventions must be conscious of “on-target” effects on non-diseased cells. This creates an extra-layer of complexity when assessing the efficacy and clinical applicability of candidate drug targets and increases the need to target mechanisms specific to disease.

**TABLE 5:** Overview of preclinical and clinical efforts targeting mitochondrial biology (as covered in this review). BCL-2 (B-cell lymphoma 2), DRP1 (dynamin-related protein 1), ER (endoplasmic reticulum), ERK (extracellular signal-regulated kinase), ETBR (endothelin B receptor), GSK3β (glycogen synthase kinase-3 beta), HMGB1 (high-mobility group box 1), HD (Huntington’s disease), IAPs (inhibitor of apoptosis proteins), MCU (mitochondrial calcium uniporter), S6K1(p70 S6 ribosomal kinase).

Process	Indication	Compound/Drug	Mechanism of action	Citation
Mitochondrial morphology	Hypoxic-ischemic encephalopathy	Sovateltide (PMZ-1620; IRL-1620)	Agonism of ETBR to promote higher differentiation of neuronal progenitor cells with improved mitochondrial morphology	NCT05514340
	Alzheimer’s disease	PD98058, mdivi-1, probucol	Inhibition of ERK to restores mitochondrial fusion/fission balance	Gan et al. (2014)
	Pulmonary hypertension	Trimetazidine	Inhibition of fatty acid oxidation to improve mitochondrial fusion/fission balance	NCT02102672
	Myocardial ischemia reperfusion injury	Astragaloside IV derivative (LS-102)	Inhibition of GSK3β-mediated DRP1 phosphorylation at S616 to reduce mitochondrial fragmentation	Chen et al. (2020)
Cellular apoptosis	Solid malignancies (patients did not respond to standard treatemnts)	Debio 1142 in combination with Nivolumab	Antagonism of IAPs to induce apoptosis in combination with immune checkpoint inhibition	NCT04122625
	Relapsed or refractory advanced malignant tumours	TQB3909	Inhibition of BCL-2 to induce apoptosis	NCT04975204
	Glioblastoma	Piticlisib (GDC-0941) in combination with ABT-263	PI3K inhibition to inhibit BAD phosphorylation at S112 and 136 sensitising to BCL-2 inhibition	Pareja et al. (2014)
	Peritoneal carcinomatosis	Mitomycin C in combination with rapamycin	Chemotherapy with mTOR inhibition to induce apoptosis by BAD dephosphorylation via inactivation of S6K1	Song et al. (2014)
	Brain damage after ischemia-reperfusion	Dexmedetomidine	Decrease in ER stress-induced neuronal apoptosis	Zhai et al. (2019)
	Neuronal injury during brain surgery	Dexmedetomidine	Minimised neuronal injury by reducing apoptosis	NCT02878707
Ca^2+^ homeostasis and MAMs regulation	Huntington’s disease	Pridopidine (ACR16)	Agonism of Sig-1R to show promise in improving HD motor phenotypes	NCT00665223, de Yebenes et al. (2011)
	Amyotrophic lateral sclerosis	Pridopidine (ACR16)	Agonism of Sig-1R to improve speech measures	NCT04615923
	Parkinson’s disease	Ruthenium red	MCU inhibition to rescue dopaminergic neurons in pink1 deficient zebrafish	Soman et al. (2017)
ROS signalling	Graft-versus host disease	MIT-001	ROS scavenging to decrease HMGB1 expression and oxidative stress	Im et al. (2015)
	Head and neck squamous cell carcinoma patients undergoing chemoradiotherapy	MIT-001	ROS scavenging to prevent chemoradiotherapy-associated oral mucositis (caused by high ROS and excessive inflammation)	NCT04651634
	Heart failure	Nicotinamide riboside	Increase in NAD+ levels to improve mitochondrial and thus cardiac function	NCT04528004
	Breast cancer patients undergoing treatment with doxorubicin-adjuvant chemotherapy	Mitoquinone	Prevention of doxorubicin-induced oxidative stress	NCT05146843

Interestingly, the modulation of these mitochondrial mechanisms through targeting phosphorylation events has been scarce. As highlighted, the close association of altered kinase activity with disease onset and maintenance provides an opportunity to tune novel compounds towards disease tissue. In addition, the majority of phosphorylation sites on these mitochondrial regulators are still unstudied. In particular, the regulation of both mitochondrial associated membranes and mitochondrial calcium homeostasis by phosphorylation is a key area of development. Overall, the further characterisation of kinase mediated regulation of mitochondrial function will both improve our knowledge of basic cellular regulation but may also elucidate currently unknown mechanisms by which mitochondria adapt to support disease.
